# *Platycodon grandiflorus*–Derived Nanovesicles Sensitize Gastric Cancer to T Cell Killing via Ferroptosis‐Driven Membrane Mechanical Remodeling

**DOI:** 10.1002/advs.77900

**Published:** 2026-09-27

**Authors:** Jian Wu, Ruijuan Zhang, Yixin Duan, Shuqi Huo, Xiang Zhang, Yibin Xing, Lingjing Ma, Tiran Jiang, Xingyu Chen, Qingmin Sun

**Affiliations:** ^1^ Jiangsu Province Key Laboratory of Tumor Systems Biology and Chinese Medicine Jiangsu Province Hospital of Chinese Medicine Affiliated Hospital of Nanjing University of Chinese Medicine Nanjing Jiangsu China; ^2^ No.1 Clinical Medical College Nanjing University of Chinese Medicine Nanjing Jiangsu China

**Keywords:** ferroptosis, gastric cancer, *Platycodon grandiflorus*‐derived nanoparticles, T cell

## Abstract

Plant‐derived nanovesicles have emerged as promising natural nanotherapeutics for cancer treatment, yet whether they can overcome the intrinsic resistance of tumor cells to cytotoxic T‐cell‐mediated killing remains largely unexplored. Here, we show that lipid‐enriched *Platycodon grandiflorus*‐derived nanovesicles (PGNs) sensitize gastric cancer to immune attack through ferroptosis‐driven plasma membrane mechanical remodeling. Mechanistically, PGNs reprogram tumor lipid composition by coordinately suppressing the SLC7A11‐GPX4 antioxidant axis while delivering polyunsaturated fatty acids, particularly Gamma‐linolenic acid, thereby promoting glutathione depletion, lipid peroxidation, and ferroptosis. Transcriptomic analyses further revealed coordinated attenuation of malignant epithelial programs together with remodeling of membrane‐associated and glutathione metabolic pathways following PGN treatment. Fluorescence lifetime imaging microscopy and atomic force microscopy revealed concomitant increases in plasma membrane tension and cellular stiffness during ferroptosis, facilitating perforin pore formation and enhancing the susceptibility of gastric cancer cells to cytotoxic T‐cell‐mediated killing. Collectively, these findings establish a mechanistic link between ferroptosis‐driven membrane mechanical remodeling and tumor immune susceptibility, highlighting plant‐derived lipid nanovesicles as a promising natural strategy for enhancing antitumor immunity.

## Graphical abstract

Plant‐derived nanovesicles from *Platycodon grandiflorus* act as bioactive lipid delivery platforms that transport γ‐linolenic acid into gastric cancer cells. This lipid cargo promotes ferroptotic lipid peroxidation, remodels membrane mechanics, and increases tumor‐cell susceptibility to cytotoxic T‐cell attack. The study reveals a ferroptosis–mechanics–immunity axis that supports PGNs as a natural strategy for gastric cancer immunotherapy.

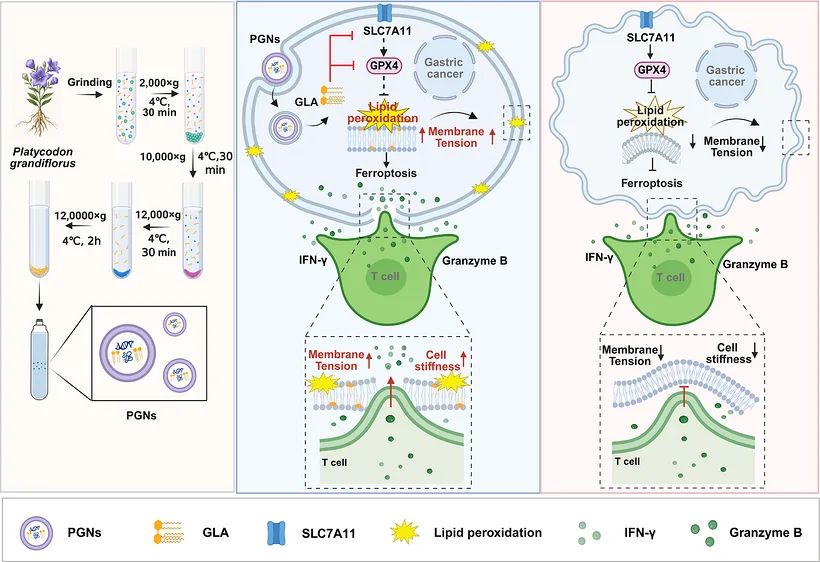

## 1 Introduction

Gastric cancer (GC) remains one of the leading causes of cancer‐related mortality worldwide, particularly in patients with advanced disease [1]. Although immune checkpoint inhibitors have improved clinical outcomes in selected patients, the overall therapeutic benefit remains limited because many tumors exhibit intrinsic resistance to cytotoxic T‐cell‐mediated killing [2]. Consequently, increasing the susceptibility of tumor cells to immune attack has become an important strategy for improving the efficacy of cancer immunotherapy.

Ferroptosis, a regulated form of cell death driven by iron‐dependent lipid peroxidation, has recently emerged as a promising approach for enhancing anti‐tumor immunity [3, 4]. Accumulating evidence indicates that activated CD8^+^ T cells promote ferroptosis by disrupting tumor antioxidant defenses, while ferroptotic tumor cells become more susceptible to immune elimination [5]. However, despite these advances, whether ferroptosis‐associated lipid remodeling directly alters the biophysical properties of the plasma membrane to regulate tumor susceptibility to cytotoxic T‐cell‐mediated killing remains largely unexplored. Elucidating this mechanistic connection may provide new opportunities for developing strategies that enhance tumor immune susceptibility.

Traditional Chinese medicine has long proposed the concept of guiding herbs (Gui‐jing), which are thought to direct co‐administered therapeutics toward specific organs or diseased sites. *Platycodon grandiflorus* was recognized as a representative guiding herb and was widely described as promoting the delivery of therapeutic agents to the lesion site [6]. Its major bioactive saponins, particularly platycodin D, exhibited anti‐inflammatory, anti‐tumor, and antioxidant activities [7]. Platycodin D also functioned as an immunological adjuvant and enhanced antigen‐specific cellular and humoral immunity with a balanced Th1/Th2 response [8]. Nevertheless, despite longstanding claims of selective lesion tropism, the molecular basis underlying its targeting properties remained poorly understood. Recent advances in herbal medicine‐derived nanovesicles (HMDNVs) have provided a tractable framework for revisiting the traditional concept in modern biological terms [9]. HMDNVs have emerged as natural nanoscale delivery systems with favorable biocompatibility, carrying diverse cargos including lipids, proteins, and nucleic acids, and remaining relatively stable in gastrointestinal environments [10, 11]. More recently, plant‐derived nanoparticles were shown to potentiate anti‐PD‐L1 efficacy through lipid‐centered metabolic rewiring, underscoring the translational relevance of plant nanostructures in cancer immunotherapy [12]. These properties provide a plausible basis for tissue delivery and immune modulation by herbal materials. Importantly, membrane lipid composition and redox homeostasis were key determinants of tumor susceptibility to immune attack. Lipid‐enriched plant‐derived vesicles therefore may provide an underexplored route to connect metabolic perturbation with anti‐tumor immunity.

Here, we show that lipid‐rich *Platycodon grandiflorus*‐derived nanovesicles (PGNs) induce a ferroptosis‐sensitive state characterized by accumulation of lipid peroxidation in GC cells. Single‐cell transcriptomic analysis further indicates that PGNs drive transcriptional programs consistent with plasma membrane remodeling. Consistently, fluorescence lifetime imaging microscopy (FLIM) and atomic force microscopy (AFM) demonstrate ferroptosis‐dependent increases in membrane tension and cellular stiffness, suggesting that ferroptotic lipid peroxidation translates oxidative damage into biophysical changes in membrane properties. Functionally, GC cells pretreated with PGNs become more susceptible to T cell‐mediated cytotoxicity. Together, these findings indicate that PUFA‐rich PGNs initiated ferroptosis and lipid peroxidation‐driven membrane remodeling, thereby enhancing anti‐tumor T cell killing and establishing a natural nanoplatform‐based strategy for ferroptosis‐enabled immune sensitization.

## 2 Results

### 2.1 PGNs Are a Class of Nanovesicles with Potent Anti‐GC Activity

**FIGURE 1:**
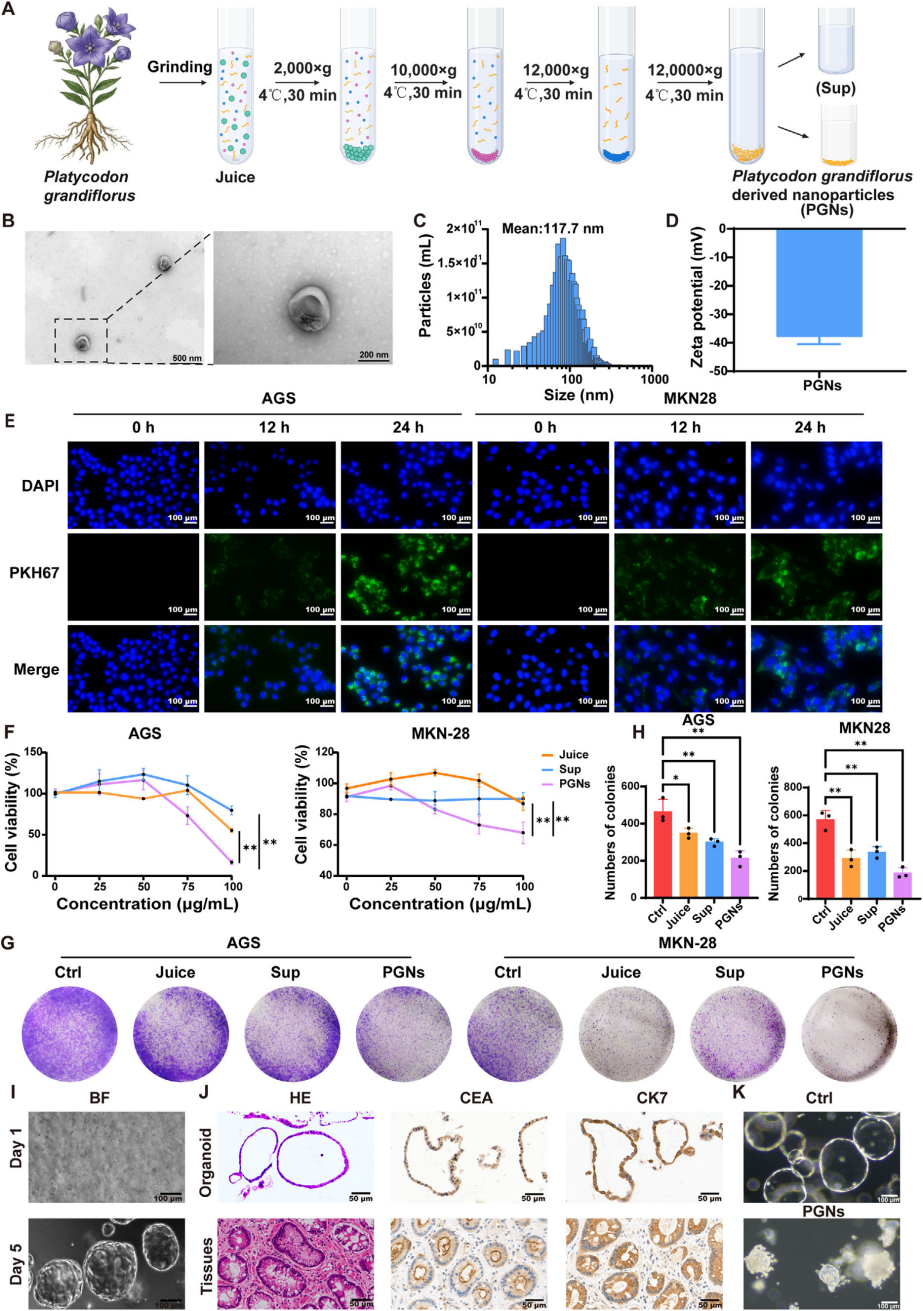
PGNs were nanovesicles with potent anti‐tumor activity. (A) Schematic representation of PGNs. (B) Representative TEM images of PGNs. Scale bar = 200 nm. (C) NTA profiles showing the particle size distribution of PGNs. (D) Zeta potential distribution of PGNs. (E) Fluorescence microscopy images showing PGNs labeled with PKH67 (green) and nuclei stained with DAPI (blue). Scale bar = 100 µm. (F) Cell viability was assessed by MTT assay in GC cells following 24 h of treatment with increasing concentrations of juice, sup, and PGNs. (G) Colony formation assay was used to evaluate cell proliferation capacity in GC cells treated with juice, Sup, and PGNs for 24 h. (H) Quantification of colony formation. (I) Patient‐derived GC organoids were cultured for 5 days and imaged by bright‐field (BF) microscopy. Scale bar = 100 µm (J) Representative H&E staining and immunohistochemical analysis showing expression of the GC markers CK7 and CEA. Scale bar = 50 µm. (K) Representative images of GC organoid growth following PGNs exposure. Scale bar = 100 µm. Data were presented as mean ± SD. Statistical significance was assessed using one‑way ANOVA with Tukey's post hoc test for single‑factor experiments, and two‑way ANOVA with Sidak's multiple‑comparisons test for comparisons between different groups at each concentration level. *p < 0.05, **p < 0.01; ns, not significant.

To isolate and characterize PGNs, fresh roots were homogenized to obtain *Platycodon grandiflorus* juice, which was subsequently subjected to ultracentrifugation to isolate PGNs from the pellet fraction (Figure 1A). TEM revealed that PGNs exhibited a typical extracellular vesicle‐like morphology, with a cup‐shaped appearance enclosed by a bilayer membrane (Figure 1B). NTA further demonstrated that PGNs had a mean diameter of 117.7 nm (Figure 1C), while zeta potential analysis revealed a surface charge of −34.88 ± 0.81 mV (Figure 1D). These physicochemical features are consistent with previously reported plant‐derived nanovesicles, supporting their structural integrity. Agarose gel electrophoresis indicated that the nucleic acid cargo of PGNs consisted predominantly of small RNAs, and SDS‐PAGE analysis showed that their major protein components were mainly distributed between 10 and 100 kDa (Figure S1A,B). Quantification by BCA assay showed that freshly isolated PGNs contained a total protein concentration of approximately 5.0 mg mL^−1^, and their purity, assessed by the particle‐to‐protein ratio, reached approximately 2 × 10^1^ ^2^ particles mg^−^^1^ protein (Figure S1C,D). To further assess the storage stability of PGNs, particle size, zeta potential and SDS‐PAGE were measured immediately after isolation and after storage at 4°C, 25°C, or −80°C for two weeks. Compared with freshly isolated PGNs, storage at −80°C most effectively preserved their physicochemical stability, whereas storage at 25°C resulted in the greatest degree of instability (Figure S1E–H). To trace the cellular uptake of PGNs, we labeled PGNs with the green fluorescent dye PKH67 and incubated them with AGS and MKN‐28 cells. A time‐dependent increase in intracellular green fluorescence was observed in both cell lines, indicating efficient internalization of PGNs. Notably, strong fluorescence signals were still detectable up to 24 h after incubation, suggesting sustained intracellular retention of PGNs (Figure 1E).

We next evaluated the anti‐tumor activity of PGNs in comparison with crude juice and supernatant (Sup) fractions. Among the three, PGNs exhibited the most pronounced inhibitory effect, suppressing cell proliferation and inducing cell death in a dose‐dependent manner in both AGS and MKN‐28 cells, with AGS cells showing greater sensitivity (Figure 1F). Consistent with these findings, PGNs markedly impaired clonogenic growth, reduced EdU incorporation, and inhibited cell migration in both cell lines (Figure 1G,H; Figure S1I–L). To further validate the anti‐tumor activity in a clinically relevant model, we established patient‐derived GC organoids. By day 5, bright‐field imaging revealed the formation of compact and cohesive solid epithelial clusters, and histologically, the organoids retained the tumor‐specific glandular architecture of the corresponding primary lesions and expressed the GC markers cytokeratin 7 (CK7) and carcinoembryonic antigen (CEA) (Figure 1I,J). Importantly, treatment with PGNs for 24 h markedly suppressed organoid growth (Figure 1K). Collectively, these results demonstrate that PGNs exert potent anti‐tumor activity.

### 2.2 PGNs Suppress Tumor Growth In Vivo with Favorable Biosafety

**FIGURE 2:**
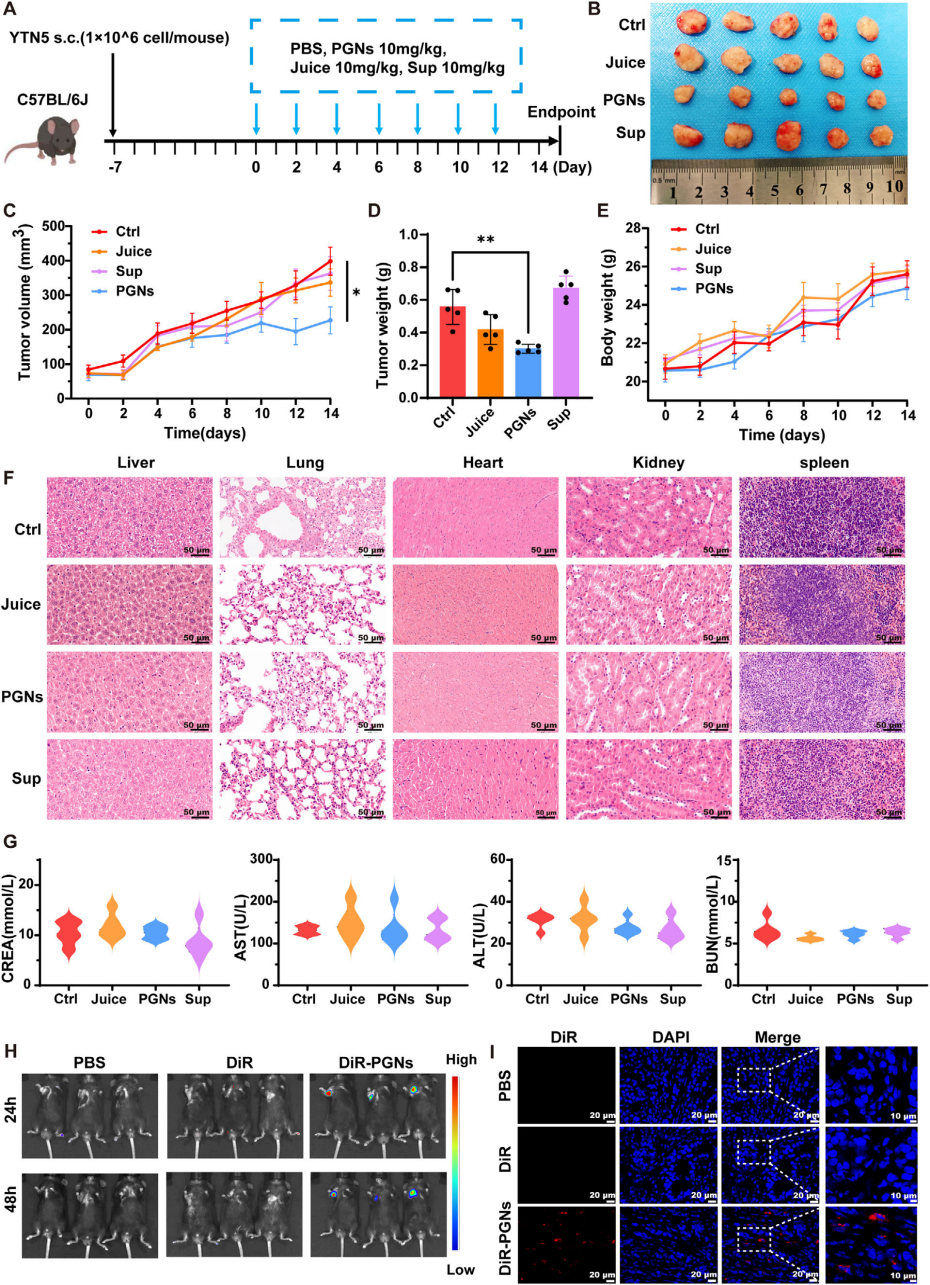
PGNs suppressed tumor growth in vivo with favorable biosafety. (A) Schedule of experimental design in C57BL/6J mouse subcutaneous model. (B) Representative images of dissected tumor from each group. (C) Tumor volumes were measured every 2 d in Ctrl, juice, Sup, and PGNs groups. (D) Tumor weights of mice in the indicated groups. (E) Body weight changes of mice in each group. (F) HE staining of lung, liver, heart, kidney, and spleen tissues from the mice receiving each treatment (scale bar = 50 µm). (G) Biochemical analysis of serum markers: AST, ALT (liver function), BUN, and CREA (kidney function) in each group. (H) Representative in vivo fluorescence images acquired at 24 and 48 h after injection of DiR‐labeled PGNs, showing sustained retention of PGNs at the tumor site (*n* = 3). (I) Confocal microscopy images of frozen tumor sections collected at 48 h after injection, showing DiR signals within the tumor parenchyma. DiR‐labeled PGNs are shown in red, and nuclei were counterstained with DAPI (blue). Scale bars = 20 µm for the overview images and 10 µm for the magnified insets. Data were presented as mean ± SEM. Statistical significance was determined using one‐way ANOVA followed by Tukey's post hoc test. Tumor growth curves were analyzed using two‐way ANOVA followed by Sidak's multiple‐comparisons test. *p < 0.05, **p < 0.01; ns, not significant.

To assess the in vivo anti‐tumor efficacy of juice, sup, and PGNs, C57BL/6J mice were subcutaneously inoculated with YTN5 cells and subsequently treated with the indicated preparations (Figure 2A). Compared with the Ctrl group, PGNs treatment markedly suppressed tumor growth, whereas juice and sup treatment showed no obvious inhibitory effect on tumor progression (Figure 2B). Accordingly, both tumor volume and tumor weight were significantly reduced in the PGNs‐treated group compared with the Ctrl group (Figure 2C,D). Notably, no significant changes in body weight were observed throughout the treatment period in mice receiving juice, sup, or PGNs, indicating favorable systemic tolerability (Figure 2E).

To further evaluate the in vivo biosafety and tumor retention of PGNs, major organs and serum samples were collected for histological, biochemical, and fluorescence‐tracking analyses. H&E staining revealed no overt pathological abnormalities in the heart, liver, spleen, lung, or kidney across all treatment groups (Figure 2F). Consistently, serum biochemical analyses showed that representative indicators of hepatic and renal function, including aspartate aminotransferase (AST), alanine aminotransferase (ALT), creatinine (CREA), and blood urea nitrogen (BUN), remained within the normal range after administration of juice, supernatant, or PGNs (Figure 2G), indicating a favorable systemic biosafety profile. Moreover, to assess the intratumoral retention of PGNs following local administration, DiR‐labeled PGNs were intratumorally injected into tumor‐bearing mice. Whole‐body fluorescence imaging showed that fluorescence signals remained predominantly confined to the tumor site at both 24 and 48 h after injection (Figure 2H). Fluorescence imaging of tumor cryosections further confirmed the presence of DiR‐labeled PGNs within tumor tissues (Figure 2I). These results demonstrate sustained intratumoral retention of PGNs, supporting the local delivery strategy used in the subsequent in vivo therapeutic and mechanistic studies.

### 2.3 PGNs Exhibit a Distinct Lipid‐Enriched Metabolic Profile Centered on Unsaturated Fatty Acids

**FIGURE 3:**
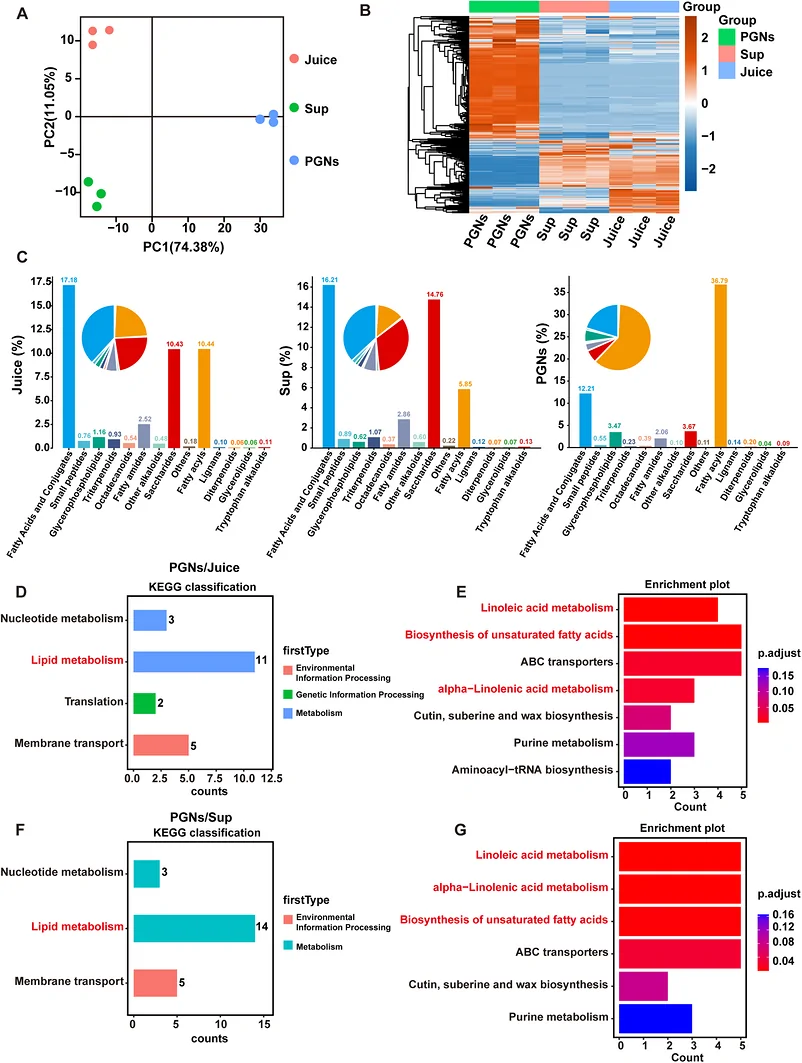
PGNs exhibited a distinct lipid‐enriched metabolic profile centered on unsaturated fatty acids. (A) PCA and (B) heatmap with hierarchical clustering of metabolite abundance profiles in juice, Sup, and PGNs. (C) Pie charts showing the proportional distribution of metabolite classes in the three groups. (D) KEGG pathway enrichment analysis of differentially abundant metabolites in PGNs compared with juice. (E) Top lipid‐associated KEGG pathways enriched in PGNs versus juice. (F) KEGG pathway enrichment analysis of differentially abundant metabolites in PGNs compared with Sup. (G) Top lipid‐associated KEGG pathways enriched in PGNs versus Sup. Combined univariate and multivariate statistical criteria were used to identify differential metabolites between groups. In the OPLS‐DA model, metabolites with a variable importance in projection (VIP) score > 1.0 and a fold change (FC) > 2 were considered significantly altered.

To identify the bioactive components responsible for the anti‐tumor activity of PGNs, non‐targeted metabolomics was performed on juice, sup, and PGNs. A total of 845 metabolites were detected and annotated across all samples. Representative total ion chromatograms acquired in both positive and negative ion modes under the established chromatographic and mass spectrometric conditions were shown in Figure S2A–F. Unsupervised principal component analysis (PCA) revealed a clear separation among the three fractions, indicating distinct metabolic profiles (Figure 3A). Consistently, hierarchical clustering further demonstrated marked differences between PGNs and the other two fractions, as illustrated by the heatmap and volcano plots (Figure 3B; Figure S2G,H). Metabolite class distribution analysis further revealed clear compositional differences. Juice and sup exhibited broadly similar profiles, dominated by fatty acids and conjugates and saccharolipids. In contrast, PGNs displayed a distinct lipid signature, characterized by marked enrichment of fatty acyls (36.79%) and a relative increase in glycerophospholipids, while retaining a comparable proportion of fatty acids and conjugates (Figure 3C). These findings indicated that PGNs were selectively enriched in lipid‐related molecules, particularly fatty acyl species, which are central determinants of membrane organization and mechanics, as the composition and unsaturation of fatty acyl chains critically shape bilayer packing, fluidity, thickness, elasticity, and curvature [13]. This enrichment suggests that PGNs may possess heightened membrane‐interacting and membrane‐remodeling capacities.

To further explore the functional relevance of these metabolic differences, KEGG pathway enrichment analysis was performed. Compared with juice and sup, the differentially abundant metabolites in PGNs were predominantly enriched in lipid metabolism‐related pathways, with the most prominent enrichment observed in linoleic acid metabolism, biosynthesis of unsaturated fatty acids, and α‐linolenic acid metabolism (Figure 3D–G). Collectively, these results identify PGNs as a metabolically distinct, lipid‐enriched fraction and provide a biochemical basis‐centered on unsaturated fatty acid metabolism‐for their enhanced biological activity.

### 2.4 PGNs Induce Ferroptosis in GC Cells by Driving Lipid Peroxidation and Redox Imbalance

Given the enrichment of unsaturated fatty acids and the metabolic connection to linoleic acid metabolism, we next treated GC cells with PGNs‐L or PGNs‐H to investigate whether PGNs induce ferroptosis. PGNs increased intracellular ROS levels in a dose‐dependent manner (Figure S3A–D). Consistently, MDA, a canonical end‐product of lipid peroxidation, was significantly increased in both MKN‐28 and AGS cells following PGNs‐H treatment relative to the Ctrl and PGNs‐L groups (Figure S3E). In parallel, intracellular GSH levels were reduced in both PGNs‐L and PGNs‐H groups, with the most pronounced depletion observed under PGNs‐H treatment (Figure S3F). To directly evaluate lipid peroxidation, cells were stained with BODIPY 581/591 C11, an oxidation‐sensitive lipid peroxidation probe. The results showed a marked increase in lipid peroxidation‐positive cells after PGNs treatment, with the most pronounced effect observed in the PGNs‐H group (Figure S3G–J). Moreover, the ferroptosis‐associated proteins GPX4 and SLC7A11 were markedly downregulated after PGNs exposure (Figure S3K,L), collectively indicating activation of a ferroptotic cell death program in GC cells.

**FIGURE 4:**
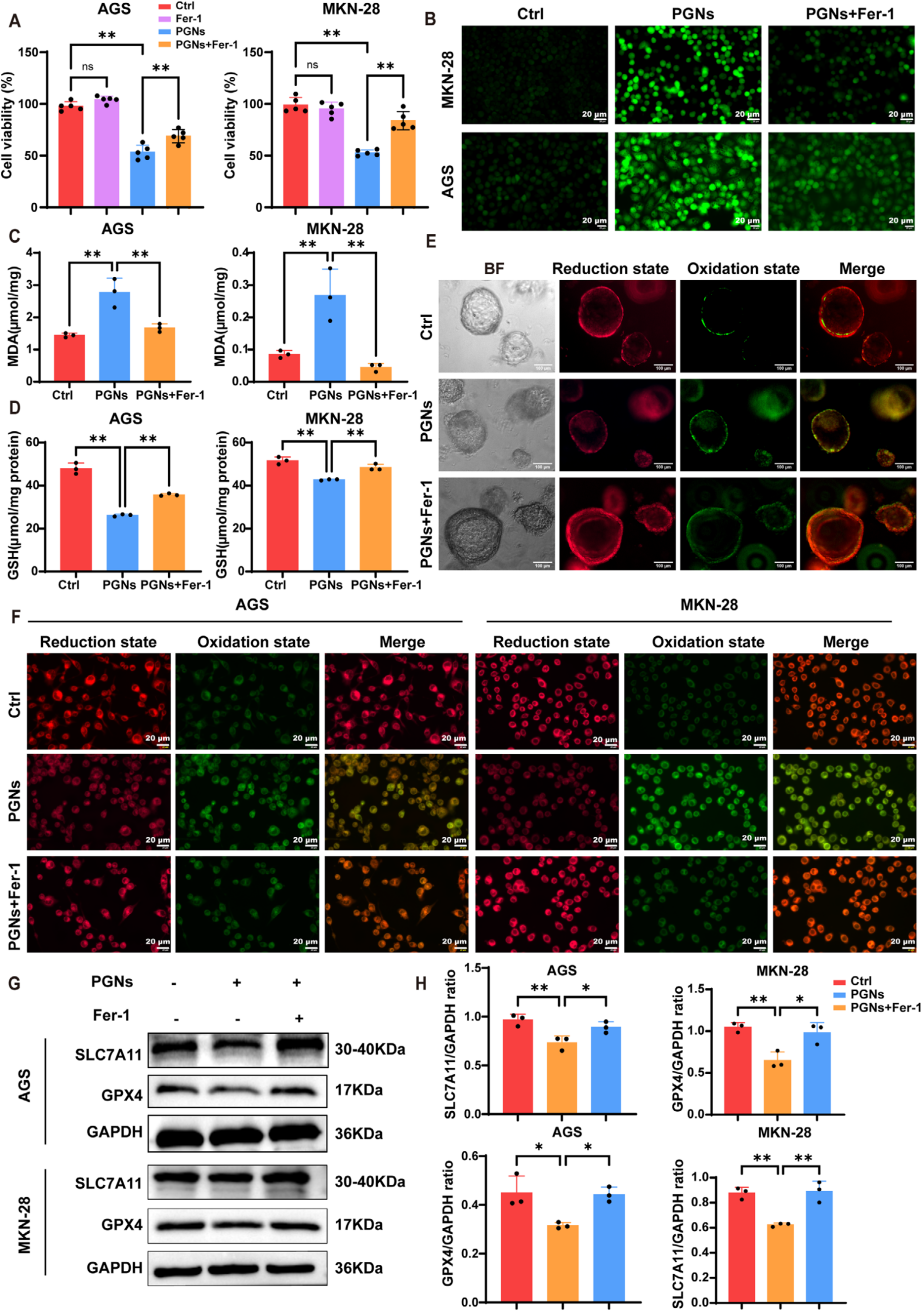
PGNs induced ferroptosis in GC cells by driving lipid peroxidation and redox imbalance. (A) AGS and MKN‐28 cells viability following PGNs treatment with or without co‐incubation of the Fer‐1. (B) Fluorescence microscopy images showing ROS levels in GC cells treated with PGNs and PGNs+Fer‐1. Scale bar = 20 µm. (C) MDA levels in cells treated with PGNs and PGNs+Fer‐1. (D) Intracellular GSH levels in cells treated with PGNs and PGNs+Fer‐1. (E) Fluorescence staining of the lipid peroxidation sensor BODIPY 581/591 C11 in GC organoids treated with PGNs in the presence or absence of Fer‐1. Scale bar = 100 µm. (F) Fluorescence staining of the lipid peroxidation sensor BODIPY 581/591 C11 in GC cells treated with PGNs in the presence or absence of Fer‐1. Scale bar = 20 µm. (G) Western blot analysis of the ferroptosis defense proteins SLC7A11 and GPX4 in GC cells treated with PGNs ± Fer‐1. (H) Densitometric quantification of SLC7A11 and GPX4 expression normalized to GAPDH. Data were presented as mean ± SD. Statistical significance was determined using one‐way ANOVA followed by Tukey's post hoc test. *p < 0.05, **p < 0.01; ns, not significant.

To further verify that PGNs‐induced cytotoxicity was mediated by ferroptosis, the selective ferroptosis inhibitor Fer‐1 was applied. PGNs‐H markedly reduced cell viability, and this effect was substantially rescued by co‐treatment with Fer‐1 (Figure 4A). Consistently, Fer‐1 attenuated the PGNs‐induced increase in intracellular ROS (Figure 4B; Figure S4A–C). In addition, Fer‐1 abrogated PGNs‐H–induced MDA accumulation and replenished depleted GSH levels (Figure 4C,D). Using C11‐BODIPY 581/591 staining, we further assessed lipid peroxidation in GC cells and GC organoids. The results showed that PGNs treatment significantly increased lipid peroxidation levels in GC cells and GC organoids, whereas co‐treatment with Fer‐1 markedly attenuated this effect (Figure 4E,F; Figure S4D–G). Similarly, Fer‐1 restored the expression of SLC7A11 and GPX4, which had been suppressed by PGNs‐H (Figure 4G,H). Collectively, these results demonstrate that PGNs induce ferroptosis‐dependent cytotoxicity in GC cells and organoids by driving lipid peroxidation and redox imbalance.

### 2.5 PGNs Potentiate T Cell Cytotoxicity by Coupling Ferroptosis with Membrane Mechanical Remodeling

**FIGURE 5:**
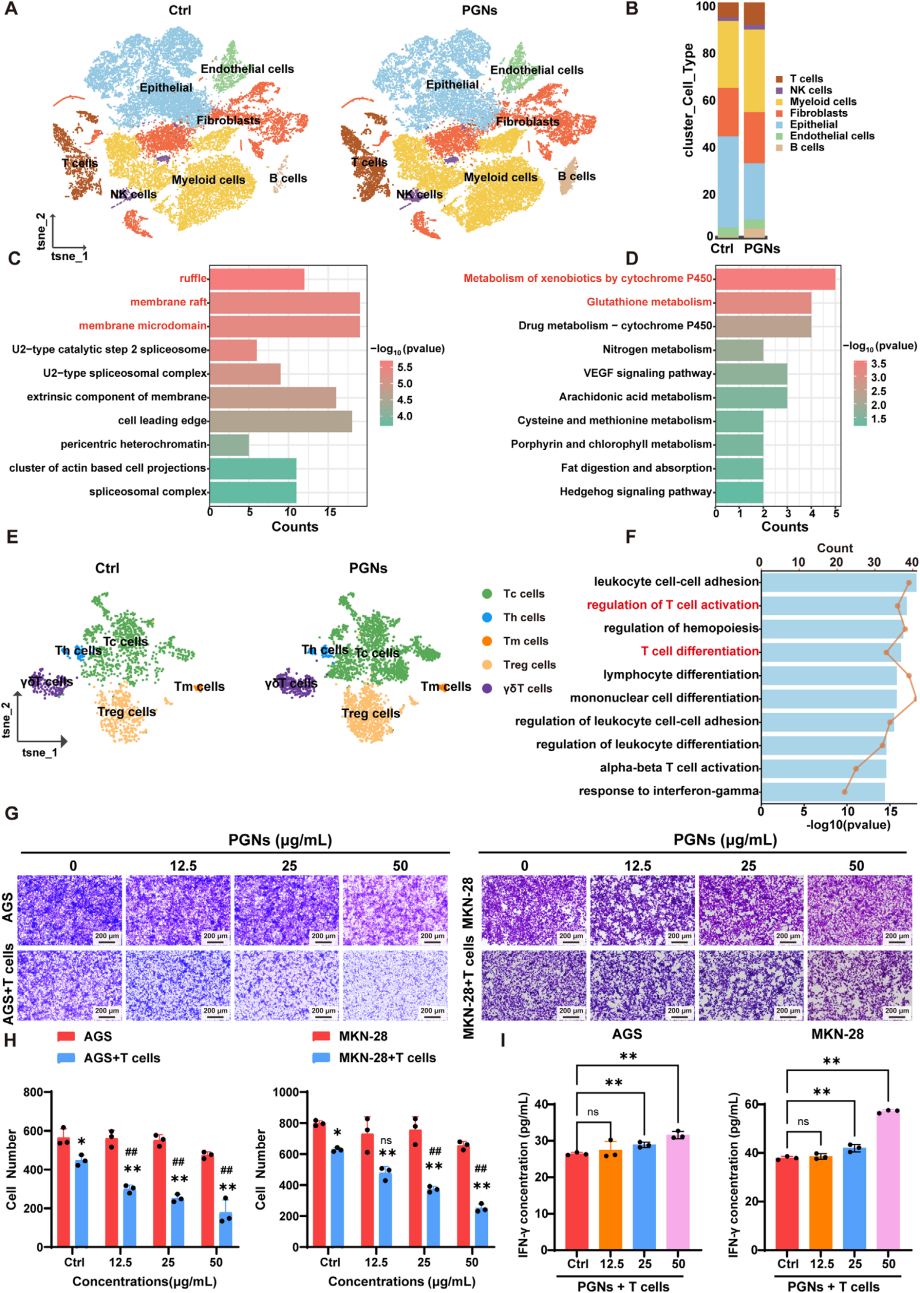
PGNs treatment attenuates malignant epithelial features and reprograms redox and membrane dynamics. (A) t‐SNE plot of single‐cell RNA sequencing data from tumor tissues collected from Ctrl and PGN‐treated mice, with three independent biological samples per group (*n* = 3 per group). (B) The proportion of each cell type in Ctrl groups and PGNs groups. (C) GO enrichment analysis of cell component for up‐regulated genes in malignant cells after PGNs treatment. (D) KEGG enrichment analysis of down‐regulated genes in malignant cells after PGNs treatment. (E) The t‐SNE projection of T cells subclusters in Ctrl group and PGNs group. (F) GO enrichment analysis of biological processes for differentially expressed genes in T cells between the two groups. (G) AGS or MKN‐28 cells were treated with increasing concentrations of PGNs and then co‐cultured with T cells, followed by crystal violet staining. (H) Quantification of crystal violet staining. (I) IFN‐γ levels in the supernatant of T cells co‐cultured with PGNs‐treated GC cells. Data were presented as mean ± SD. Statistical significance was assessed using one‑way ANOVA with Tukey's post hoc test for single‑factor experiments, and two‑way ANOVA with Sidak's multiple‑comparisons test for comparisons between different groups at each concentration level. The interaction between concentration and group was evaluated within the two‑way ANOVA model. *, #*p* < 0.05; **, ##*p* < 0.01; ns, not significant. * indicates between‐group comparisons at the same concentration, whereas # indicates within‐group comparisons across different concentrations.

Single‐cell RNA sequencing was performed on untreated and PGNs‐treated tumor tissues from C57BL/6J mouse models to further investigate the tumor‐suppressive mechanisms of PGNs. Unsupervised clustering identified seven major cell populations based on canonical marker genes, including B cells, endothelial cells, epithelial cells, fibroblasts, myeloid cells, NK cells, and T cells (Figure 5A; Figure S5A). Cellular composition analysis revealed that PGNs treatment markedly increased immune cell infiltration, as evidenced by elevated proportions of myeloid cells, T cells, and B cells, together with a concomitant reduction in epithelial cells relative to Ctrl tumor (Figure 5B). To further assess the impact of PGNs on tumor epithelial cells, copy number variation (CNV) analysis was performed to distinguish malignant from non‐malignant epithelial populations (Figure S5C,D). InferCNV analysis enabled the identification of malignant epithelial cells within the tumor tissues (Figure S5B). This result was further supported by the expression patterns of classical malignant epithelial cell marker genes (Figure S5E). Notably, PGNs treatment significantly reduced CNV scores in these malignant epithelial cells (Figure S5F). GO analysis showed that upregulated genes were predominantly enriched in cellular component terms related to membrane dynamics, including ruffles, membrane rafts, membrane microdomains, and the extrinsic component of the plasma membrane (Figure 5C), suggesting that PGNs treatment induces prominent membrane remodeling. In parallel, GO and KEGG analyses of globally downregulated genes revealed significant enrichment of pathways associated with glutathione transferase activity and glutathione‐dependent detoxification processes (Figure 5D; Figure S5G), indicating disrupted redox homeostasis. These findings demonstrate that PGNs treatment attenuates malignant epithelial features and reprograms membrane‐and redox‐associated pathways, thereby providing mechanistic insight into its anti‐tumor activity.

T cells were further classified into five subpopulations, including cytotoxic T lymphocytes (Tc), T helper cells (Th), memory T cells (Tm), regulatory T cells (Treg), and γδ T cells (Figure 5E; Figure S6A). Pseudotime trajectory analysis revealed a differentiation path originating from Treg cells and diverging into two terminal branches, namely γδ T cells and Tc cells. Notably, T cells from the PGNs‐treated group exhibited a pronounced bias toward the Tc cell fate, suggesting enhanced tumor‐killing potential (Figure S6B,C). To further characterize the functional state of T cells, we established an in vitro co‐culture system of T cells and GC cells to assess T cell‐mediated cytotoxicity. PGNs treatment markedly enhanced T cell–dependent killing of GC cells (Figure 5G,H; Figure S6F). Consistently, CBA analysis showed a dose‐dependent increase in IFN‐γ levels in the co‐culture supernatants following PGNs treatment (Figure 5I), and flow cytometric analysis further confirmed increased Granzyme B expression in CD8^+^T cells (Figure S6D,E). Notably, the enhanced GC cell killing was largely abolished by the Fer‐1, indicating that ferroptosis contributes to the PGNs‐mediated augmentation of T cell cytotoxicity (Figure S6G).

To further characterize the effect of PGNs on tumor‐infiltrating T cells, we reanalyzed the T‐cell compartment in the scRNA‐sequencing dataset. Compared with the Ctrl group, PGNs treatment increased the proportion of effector and proliferating T cells while reducing exhausted T cells, accompanied by decreased expression of exhaustion‐associated genes, including Tnfrsf18, Pdcd1, and Havcr2, whereas Lag3 expression remained unchanged (Figure S7A–D). To determine whether PGNs directly affected T‐cell function, activated T cells were treated with PGNs in vitro. PGNs did not alter T‐cell viability, the proportion of CD8^+^ T cells, or CD25 expression under either low‐ or high‐dose conditions, indicating that PGNs did not impair T‐cell survival or activation (Figure S7E–K). Consistently, TNF‐α production remained unchanged, whereas high‐dose PGNs significantly increased IFN‐γ secretion (Figure S7L,M). Together, these findings indicate that PGNs alleviate T‐cell exhaustion within the tumor microenvironment without compromising T‐cell viability or activation and may further enhance T‐cell effector function.

**FIGURE 6:**
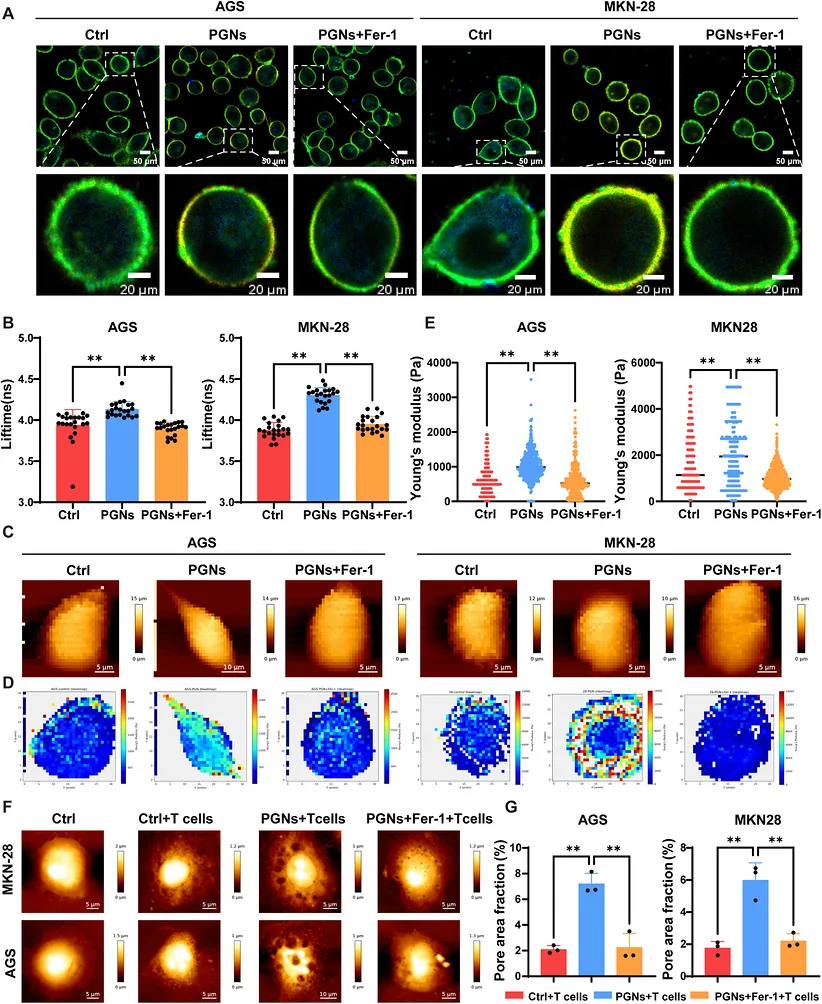
PGNs induced ferroptosis and increased membrane tension in GC Cells. (A) Representative FLIM images of Flipper‐TR‐labeled plasma membranes in GC cells treated with PGNs in the presence or absence of Fer‐1. Scale bar = 20 µm (B) Quantification of Flipper‐TR fluorescence lifetime measured by FLIM. (C) AFM measurement of cell stiffness in GC cells treated with PGNs in the presence or absence of Fer‐1. Scale bar = 5 µm. (D) Representative AFM stiffness heatmaps of GC cells. (E) Quantification of the Young's modulus of GC cells measured by AFM. (F) AFM analysis of membrane pore formation in GC cells after 12 h of co‐culture with T cells following PGNs treatment in the presence or absence of Fer‐1. Scale bar = 5 µm. (G) Quantification of the proportion of pore area formed on the cell membrane. Data were presented as mean ± SD. Statistical significance was determined using one‐way ANOVA followed by Tukey's post hoc test. *p < 0.05, **p < 0.01; ns, not significant.

Given that ferroptotic cells are characterized by increased membrane tension, which has been shown to facilitate T cell‐mediated cytotoxicity, FLIM in combination with the membrane tension‐sensitive probe Flipper‐TR was employed to assess membrane tension in GC cells. Compared with the Ctrl group, PGNs‐treated cells exhibited a significant increase in fluorescence lifetime (τ), indicating elevated local membrane tension driven by PGNs‐induced lipid peroxidation, and this increase was notably reversed by Fer‐1 (Figure 6A,B). AFM measurements showed that PGNs treatment significantly increased the stiffness of AGS and MKN‐28 cells, indicating a ferroptosis‐dependent mechanical remodeling process (Figure 6C–E). Furthermore, AFM‐based visualization of membrane pores at the GC cell‐immune cell interface revealed enhanced pore formation during co‐culture with T cells after PGNs treatment, as reflected by both an increased pore incidence and enlarged pore size compared with Ctrl conditions. Importantly, PGNs‐induced changes in pore formation were largely abolished by Fer‐1 (Figure 6F,G). Collectively, these data demonstrate that PGNs potentiate T cell cytotoxicity by coupling ferroptosis with membrane mechanical remodeling.

### 2.6 GLA Recapitulates the Ferroptosis‐Dependent Anti‐Tumor and Immune Potentiating Effects of PGNs

**FIGURE 7:**
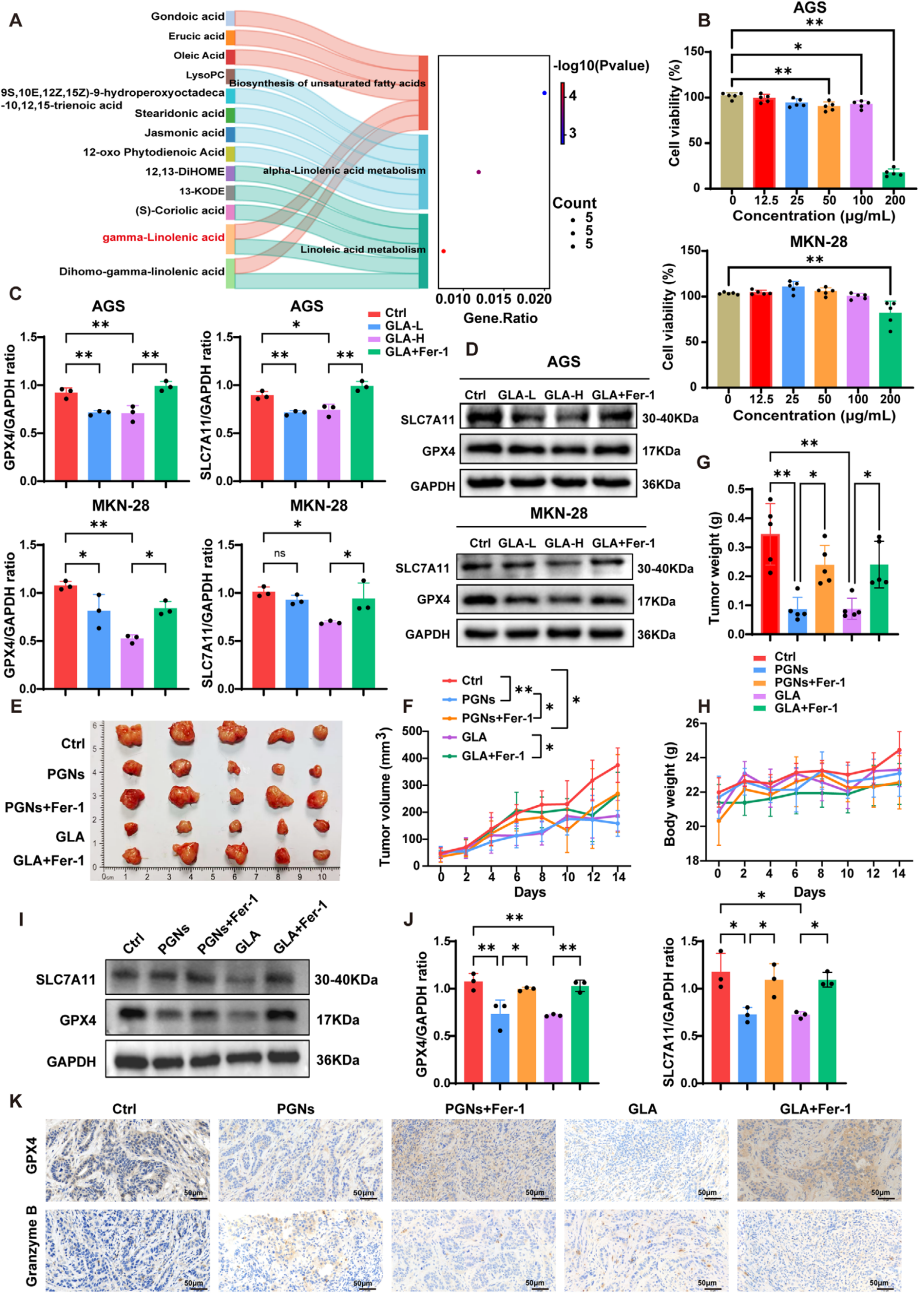
PGNs induced ferroptosis via GLA and promoted T cell mediated cytotoxicity to suppress tumor growth. (A) Lipid metabolism‐related metabolites in PGNs. (B) MTT analysis of GC cell viability after co‐incubation with different concentrations of GLA. (C) Quantification of protein expression. (D) Western blot analysis of SLC7A11 and GPX4 in GLA‐treated GC cells in the presence or absence of Fer‐1. (E) Representative images of excised tumors collected at the experimental endpoint from mice bearing syngeneic tumors treated with PGNs or GLA alone or in combination with Fer‐1. (F) Tumor volume and tumor weight (G) in mice treated with PGNs or GLA alone or in combination with Fer‐1. (H) Body weight of mice monitored during treatment. (I, J) Western blot analysis of GPX4 and SLC7A11 in tumor lysates. (K) Immunohistochemical evaluation of GPX4 and granzyme B in tumor sections. Data are presented as mean ± SD. Statistical significance was determined using one‐way ANOVA followed by Tukey's post hoc test. Tumor growth curves were analyzed using two‐way ANOVA followed by Sidak's multiple‐comparisons test. *p < 0.05, **p < 0.01; ns, not significant.

Given the enrichment of lipid‐related components in PGNs, we sought to identify the bioactive lipid species responsible for the observed effects. Notably, GLA was implicated in multiple enriched lipid metabolic pathways (Figure 7A). Based on relative abundance and biochemical relevance, GLA was prioritized as a representative unsaturated fatty acid candidate. Consistent with this, treatment with GLA reduced the viability of both AGS and MKN‐28 cells in a dose‐dependent manner (Figure 7B). Western blot analysis further showed that GLA markedly decreased the expression of the ferroptosis‐associated proteins SLC7A11 and GPX4 in both cell lines, whereas co‐treatment with Fer‐1 largely restored their expression (Figure 7C,D). These findings suggest that GLA, as a representative lipid component of PGNs, contributes to PGNs‐induced ferroptotic responses in GC cells.

To further evaluate the in vivo anti‐tumor effects of PGNs and GLA, either alone or in combination with Fer‐1, a syngeneic subcutaneous tumor model was established. Treatment with PGNs or GLA alone suppressed tumor growth, whereas this effect was attenuated by co‐administration of Fer‐1 (Figure 7E–G). Throughout the treatment period, no significant differences in body weight were observed among the groups (Figure 7H). Mechanistically, PGNs or GLA markedly reduced the expression of GPX4 and SLC7A11, while Fer‐1 substantially rescued their downregulation (Figure 7I,J). Immunohistochemical analysis further confirmed reduced GPX4 staining in tumor following PGNs or GLA treatment. Notably, PGNs and GLA increased granzyme B levels. Conversely, Fer‐1 restored GPX4 expression and reduced granzyme B. These results indicate that ferroptosis contributes both to tumor injury and to enhanced cytotoxic immune effector engagement in vivo (Figure 7K). Collectively, these findings suggest that GLA, as a candidate active component of PGNs, may drive ferroptosis‐dependent tumor suppression and enhance anti‐tumor immunity in vivo.

### 2.7 PGNs Enhance Antitumor Efficacy and Promote Intratumoral CD8^+^T‐Cell Responses During PD‐1 Blockade

**FIGURE 8:**
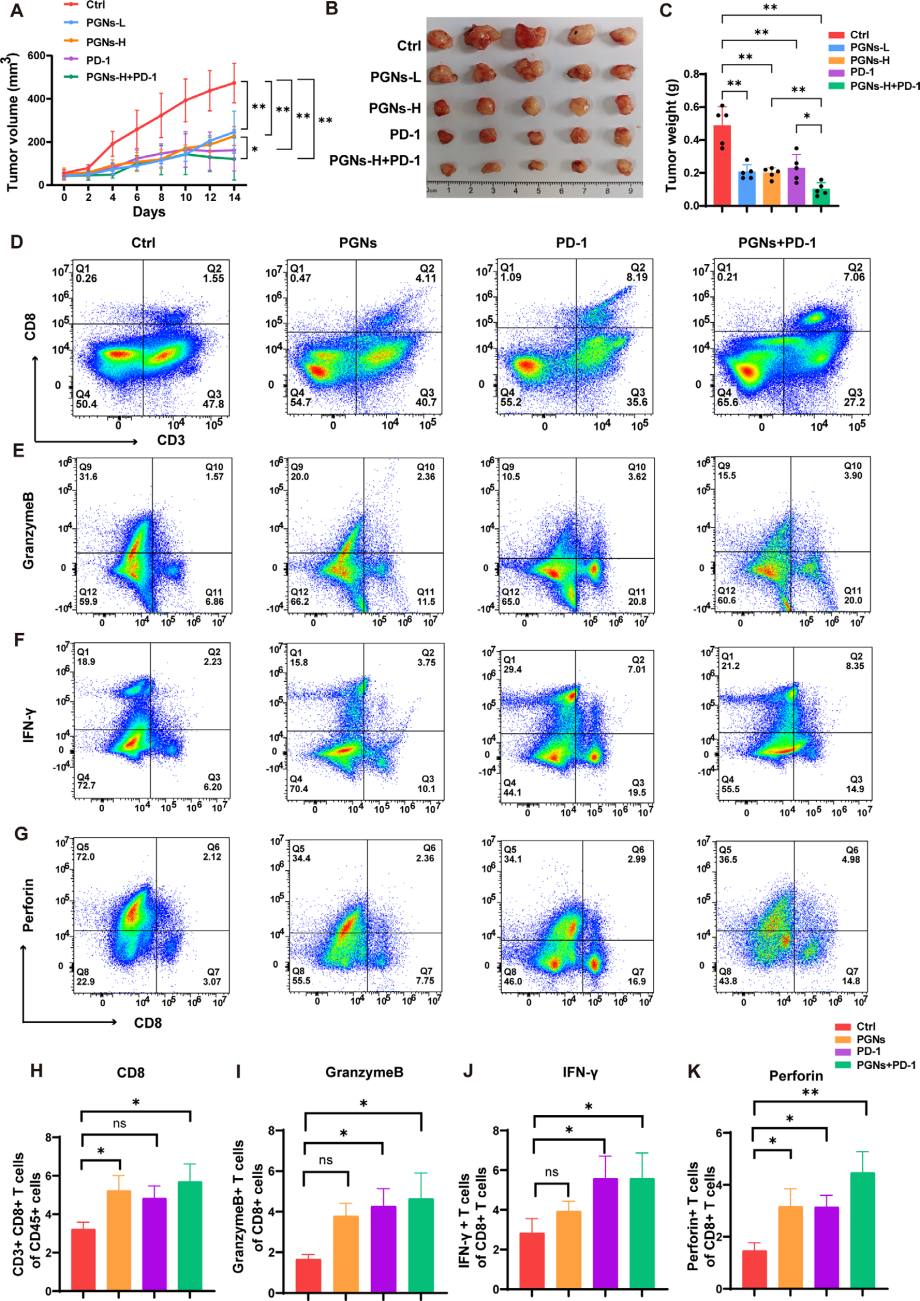
PGNs enhance the therapeutic efficacy of anti‐PD‐1 treatment and promote CTL effector activity in vivo. (A) Tumor growth curves of mice in the indicated treatment groups. (B) Representative images of excised tumors collected at the endpoint. (C) Quantification of endpoint tumor weight. (D) Flow cytometric quantification of tumor‐infiltrating CD3^+^ CD8^+^ T cells. Flow cytometric quantification of tumor‐infiltrating Granzyme B^+^ CD8^+^ T cells (E), IFN‐γ^+^ CD8^+^ T cells (F), and Perforin^+^ CD8^+^ T cells (G). (H) Quantification of CD3^+^ CD8^+^ T cells. (I) Quantification of Granzyme B^+^ CD8^+^ T cells, IFN‐γ^+^ CD8^+^ T cells (J), and Perforin^+^ CD8^+^ T cells (K) in tumor tissues from the indicated treatment groups. Data are presented as mean ± SEM; Statistical significance was determined using one‐way ANOVA followed by Tukey's post hoc test. Tumor growth curves were analyzed using two‐way ANOVA followed by Sidak's multiple‐comparisons test. *n* = 5 mice per group. *p < 0.05, **p < 0.01, ns, not significant.

To evaluate the anti ‐ tumor efficacy of PGNs alone and in combination with PD‐1 blockade, tumor‐bearing C57BL/6 mice were treated with low‐ or high‐dose PGNs (PGNs‐L or PGNs‐H), anti‐PD‐1, or PGNs‐H plus anti‐PD‐1. No significant body‐weight loss was observed during treatment, indicating favorable tolerability under the tested conditions (Figure S8A). Both PGNs and anti‐PD‐1 suppressed tumor growth compared with the Ctrl group, whereas the combination treatment resulted in the lowest overall tumor burden (Figure 8A,B). Although the difference in tumor volume between the combination and anti‐PD‐1 monotherapy groups did not reach statistical significance, endpoint tumor weight was significantly lower in the combination group than in either the PGNs‐H or anti‐PD‐1 group (Figure 8C).

We next examined tumor‐infiltrating CD8^+^ T‐cell responses by flow cytometry. PGNs treatment significantly increased the proportion of CD3^+^CD8^+^ T cells among CD45^+^ tumor‐infiltrating leukocytes and the frequency of Perforin^+^ cells within the CD8^+^ T‐cell compartment, without significantly altering the frequencies of IFN‐γ^+^ or Granzyme B^+^ CD8^+^ T cells (Figure 8D–K). In contrast, anti‐PD‐1 increased the frequencies of IFN‐γ^+^, Granzyme B^+^, and Perforin^+^ CD8^+^ T cells but did not significantly alter the overall abundance of intratumoral CD8^+^ T cells. Notably, the combination treatment was associated with increases in both intratumoral CD8^+^ T‐cell abundance and all three cytotoxic effector‐marker‐positive subsets compared with the Ctrl group (Figure 8D–K). The gating strategy was shown in Figure S8C. Together, these findings indicate that PGNs promote CD8^+^ T‐cell accumulation and complement PD‐1 blockade by supporting cytotoxic effector responses within the tumor microenvironment.

## 3 Discussion

In this study, we identified PGNs as a class of lipid‐rich natural nanocarriers that exerted potent anti‐tumor activity in GC. Mechanistically, PGNs were enriched in PUFAs, particularly GLA, and efficiently delivered these lipids into GC cells. This process drove lipid peroxidation and induced a ferroptosis‐sensitive state characterized by disruption of redox homeostasis and accumulation of peroxidized membrane lipids. Notably, our findings further demonstrated that ferroptosis induced by PGNs was not restricted to intracellular oxidative damage but was coupled to coordinated remodeling of plasma membrane architecture and mechanics.

How does PGNs‐induced ferroptosis compare with canonical ferroptosis pathways? Our results revealed both similarities to and distinctions from classical ferroptosis. Recent studies have increasingly recognized ferroptosis as a lipid‐centric form of regulated cell death governed by the balance between peroxidizable phospholipids and antioxidant defense systems [14, 15]. Consistent with this framework, our data showed that PGNs treatment suppressed glutathione‐associated metabolic programs and downregulated key components of the SLC7A11‐GPX4 axis, thereby diminishing the cellular capacity to detoxify lipid peroxides. Although ferroptosis was classically defined as iron‐dependent phospholipid peroxidation [16, 17], tumor cells often exhibited pronounced heterogeneity in antioxidant wiring. Single‐cell transcriptomic analysis revealed that this effect was not uniform across all tumor cells but instead shifted the population toward a ferroptosis‐permissive state, highlighting the intrinsic heterogeneity of tumor redox regulation. The ability of Fer‐1 to rescue both lipid peroxidation and cell death further confirmed that chain‐propagating lipid radical reactions were essential for the observed phenotype, thereby distinguishing ferroptosis from non‐specific oxidative stress. A key point to emphasize is that the ferroptosis we observed was induced by a naturally occurring PUFA (GLA) delivered via plant vesicles. This GLA‐driven ferroptosis pathway represents a distinct, metabolically mediated entry point that may offer advantages in terms of specificity and safety.

Beyond its canonical biochemical features, ferroptosis has recently been implicated in the regulation of membrane properties. Recent studies suggest that lipid peroxidation preferentially accumulates at the plasma membrane, thereby increasing membrane tension, promoting cation influx, and accelerating ferroptotic membrane damage [18, 19]. In line with these advances, our study provided direct biophysical evidence that PGNs‐induced lipid peroxidation was associated with increased membrane tension and cellular stiffness. These mechanical changes are unlikely to represent passive consequences of membrane damage alone, but instead suggest a functionally relevant form of biophysical remodeling. Thus, ferroptosis emerged not only as a metabolic and oxidative process but also as a regulator of membrane biophysical homeostasis. Our AFM and FLIM analyses further indicate that PGN‐induced ferroptosis increases plasma membrane tension and cellular stiffness, suggesting that ferroptotic lipid peroxidation is translated into a mechanically altered membrane state. Such mechanical remodeling provides a plausible mechanobiological explanation for the enhanced susceptibility of tumor cells to cytotoxic T‐cell‐mediated killing observed in this study. Consistent with previous mechanobiology studies, cytotoxic T lymphocytes generate mechanical forces at the lytic synapse, and increased membrane tension has been shown to facilitate perforin‐mediated membrane permeabilization [20]. Notably, membrane tension and stiffness alterations do not merely affect cell physical properties but also modulate mechanosensitive signaling pathways, such as YAP/TAZ, Piezo1, and integrin signaling, that may further regulate immune responses [21, 22]. This observation reinforces the functional relevance of membrane mechanics in the context of ferroptosis‐driven immune sensitization.

A key implication of this membrane remodeling lies in its impact on anti‐tumor immunity. Cytotoxic T lymphocyte‐mediated killing depends not only on biochemical signaling but also on the mechanical properties of target cells. Studies have established the immune synapse as a force‐bearing killing apparatus [20, 23]. Cytotoxic T lymphocytes actively apply mechanical forces that promote perforin pore formation and potentiate target‐cell lysis [20]. Conversely, tumor cells can evade killing by adopting a mechanically “soft” state that limits perforin‐mediated pore formation [24, 25]. The immune synapse is a force‐dependent interface, and effective perforin‐mediated pore formation requires sufficient membrane tension and structural resistance [24, 25]. Our data demonstrated that PGNs‐induced ferroptosis shifted GC cells toward a mechanically stiffened state, thereby enhancing their vulnerability to cytotoxic T lymphocyte‐mediated cytotoxicity. This was supported by increased pore formation and augmented T cell‐dependent killing following PGNs treatment, effects that were reversed upon ferroptosis inhibition. These findings established a functional link between lipid peroxidation‐driven membrane remodeling and immune effector activity. Notably, our findings support a ferroptosis‐dependent link between membrane mechanical remodeling and enhanced susceptibility to CTL‐mediated cytotoxicity. Nevertheless, the precise molecular events occurring at the immune synapse remain to be further elucidated. In particular, whether ferroptosis‐induced membrane remodeling influences immune synapse stability, perforin pore formation dynamics, or the lateral organization of immunoregulatory surface molecules such as MHC‐I and PD‐L1 warrants further investigation. Addressing these questions will provide a more comprehensive understanding of how membrane biomechanics regulate antitumor immune responses. From a translational perspective, the intratumoral administration strategy adopted in this study enabled sustained local retention of PGNs within tumors while exhibiting favorable systemic biosafety, thereby providing a rationale for the local therapeutic effects observed in vivo.

Importantly, our study positions plant‐derived nanovesicles as metabolically active lipid delivery platforms rather than passive carriers. Unlike conventional nanomedicine strategies that rely on drug encapsulation, PGNs intrinsically contain bioactive lipid species capable of reprogramming GC cell metabolism and membrane composition. The identification of GLA as a candidate bioactive lipid further supports a model in which exogenous PUFA supply expands the pool of peroxidation‐prone phospholipids, thereby lowering the threshold for ferroptotic execution. Although our findings strongly support GLA as a representative bioactive lipid contributing to the ferroptosis‐inducing activity of PGNs, the necessity of GLA cannot yet be definitively established. Selective depletion of GLA from intact PGNs without disrupting vesicle integrity or overall lipid composition remains technically challenging. Future advances in vesicle engineering and lipid editing may enable more definitive validation of the specific contribution of GLA to the biological activities of PGNs. Notably, PGNs are not merely vehicles for GLA; rather, they function as an integrated bioactive platform that delivers additional lipid species and other vesicular cargos while simultaneously remodeling the plasma membrane through vesicle–cell interactions, thereby achieving multifaceted regulation beyond simple drug delivery.

Taken together, our findings demonstrate that coupling ferroptosis induction with membrane mechanical remodeling represents a mechanistically distinct strategy to overcome a critical limitation of current immunotherapy in GC, namely the intrinsic resistance of GC cells to immune‐mediated killing. By lowering the physical and energetic barriers to immune cytotoxicity, PGNs enhance tumor susceptibility to immune attack. Moreover, compared with synthetic nanocarriers, PGNs offer several inherent advantages, including natural origin, favorable biocompatibility, renewable sources, and scalable production, making them attractive candidates for translational development.

Despite these encouraging findings, several important issues warrant further investigation to fully realize the translational potential of PGNs. Although our conclusions were supported by complementary evidence from human gastric cancer cell lines, patient‐derived organoids, and an immunocompetent syngeneic mouse model, further validation in humanized in vivo models will strengthen the translational applicability of the proposed mechanism. In addition, while our in vivo tracking experiments demonstrated sustained intratumoral retention of PGNs following local administration, future studies evaluating their pharmacokinetics, tissue biodistribution, and metabolic fate after systemic delivery will further facilitate the translational development of plant‐derived nanovesicles. Finally, although our findings support GLA as a candidate bioactive lipid contributing to the biological activity of PGNs, the complex composition of PGNs beyond GLA warrants further investigation, and the precise endocytic mechanisms governing PGN uptake remain to be fully elucidated. Addressing these questions will further facilitate the rational design and translational development of plant‐derived nanovesicles as next‐generation cancer immunotherapeutics.

## 4 Conclusions

In summary, we establish a mechanistic link between plant vesicle lipid delivery, ferroptosis induction, membrane remodeling, and T cell‐mediated cytotoxicity in GC. PGNs, enriched in the polyunsaturated fatty acid GLA, induce a ferroptosis‐permissive state characterized by single‐cell suppression of glutathione metabolism and the accumulation of ferroptotic lipid peroxidation. By directly visualizing with FLIM and AFM, and in line with single‐cell profiling that reveals coordinated membrane remodeling and enhanced T cell activation, we show that ferroptotic lipid peroxidation remodels membrane lipid composition and mechanics, thereby enhancing perforin‐mediated membrane pore formation and consequent GC cell killing. Collectively, our findings redefine ferroptosis as a biophysical vulnerability amplifier on the GC cell surface and propose edible plant‐derived nanovesicles as a bioactive therapeutic platform to increase tumor killability, thereby providing a rationale for ferroptosis‐enabled immune sensitization in gastric cancer.

## 5 Materials and Methods

### 5.1 Cell Lines

Human GC cell lines (AGS and MKN28) used in this experiment were obtained from the Cell Bank of Type Culture Collection of the Chinese Academy of Sciences (Shanghai, China). The cells were cultured in RPMI 1640 medium (Gibco, USA) supplemented with 10% fetal bovine serum (Evergreen Company, China). Mouse GC cell line YTN5 was kindly provided by Professor Sachiyo Nomura from Hoshi University, Japan. The cells were cultured in DMEM medium (Gibco, USA) supplemented with 20% fetal bovine serum and 1% penicillin–streptomycin. All cell lines were maintained at 37°C in a humidified atmosphere containing 5% CO_2_.

### 5.2 Isolation of PGNs

Fresh *Platycodon grandiflorus* roots (2 kg) were thoroughly rinsed with distilled water to remove surface contaminants. The cleaned roots were homogenized with phosphate‐buffered saline (PBS) at a 1:3 (w/w) ratio using a mechanical blender to extract cellular fluids. The homogenate was subjected to sequential differential centrifugation at 4°C. First at 2000×*g* for 30 min to remove coarse debris, followed by 10 000×*g* for 30 min and then 12 000×*g* for 30 min to eliminate residual insoluble particles. For nanoparticles isolation, the filtrate was ultracentrifuged at 120 000×*g* for 2 h at 4°C using a Type 70 Ti rotor (Beckman Coulter), yielding a pellet enriched in PGNs. The pellet was gently resuspended in PBS. All samples were stored at −80°C until further analysis.

### 5.3 Characterization of PGNs

The size distribution, concentration, and zeta potential of PGNs were characterized using nanoparticle tracking analysis (NTA) (Particle Metrix, Meerbusch, Germany). Briefly, PGNs were appropriately diluted in PBS and injected into the laser chamber via a 1 mL syringe. The morphology of PGNs was examined by transmission electron microscopy (TEM). For TEM analysis, PGNs were fixed with 2% paraformaldehyde (PFA), dried onto carbon‐coated copper grids, and stained with 1% phosphotungstic acid. After staining, the grids were air‐dried for 15 min and observed under a JEM‐2100F electron microscope (JEOL, Japan). The total protein concentration of PGNs was determined using a BCA assay following the manufacturer's instructions. The stability of PGNs was assessed by monitoring changes in their size distribution, concentration, and zeta potential after storage at 4°C, 25°C, and −80°C for two weeks, using the same NTA system.

### 5.4 Coomassie Brilliant Blue Staining

Following extraction with RIPA lysis buffer, proteins from PGNs subjected to electrophoresis on a 10% SDS‐PAGE gel (E303, Vazyme). The separation was performed initially at 80 V for 30 min, subsequently at 120 V for 1 h. The gel was then excised and stained with Coomassie Blue (G2059, Servicebio) for 2 h. The protein of the PGNs was visualized and analyzed using a gel imaging analysis system.

### 5.5 Gel Electrophoresis of Agarose

A 3% agarose gel was prepared (BS081, Biosharp), poured into the electrophoresis tank, and allowed to solidify at room temperature for 30 min. A mixture containing 5 µL of PGNs RNA sample and 5 µL RNA marker (G3371, Servicebio) were prepared, thoroughly mixed, and loaded into the sample wells. The gel was placed in the electrophoresis chamber, submerged in 1× TBE buffer, and run at 120 V for 30 min. After electrophoresis, the RNA bands were visualized and recorded using a UV gel imaging system.

### 5.6 Cellular Uptake of PGNs

To assess cellular uptake, PGNs were labeled with the fluorescent dye PKH67 (C3635S, Beyotime). AGS and MKN‐28 cells were seeded in six‐well plates and cultured overnight. Subsequently, the cells were incubated with PKH67‐labeled PGNs for 12 h or 24 h. After incubation, the cells were fixed with 4% paraformaldehyde, and the nuclei were stained with DAPI. Fluorescence images were then captured using a fluorescence microscope.

### 5.7 Cell Viability Assay

The inhibitory effect of *Platycodon grandiflorus* derived samples on GC cells was evaluated in vitro using an MTT assay. AGS, MKN‐28 cells were seeded in 96‐well plates and treated with *Platycodon grandiflorus* juice, supernatant (Sup), or PGNs at protein concentrations ranging from 25 to 100 µg mL^−1^. The cells were then co‐incubated at 37°C for 24 h. Subsequently, 10 µL of MTT working solution (ST316, Beyotime) in 100 µL of fresh medium was added to each well, and the mixture was incubated for an additional 2 h. Finally, the optical density (OD) at 490 nm was measured using a microplate reader (BioTek, USA) to determine cell viability.

### 5.8 Untargeted Metabolomic Analysis

Untargeted metabolomic analysis was performed on samples collected from the juice, sup, and PGNs groups using an LC‐MS platform. Chromatographic separation was achieved on a Waters ACQUITY Premier HSS T3 column (1.8 µm, 2.1 × 100 mm). The column temperature was maintained at 40°C, the flow rate was set at 0.30 mL min^−1^, and the injection volume was 2 µL. The mobile phase consisted of solvent A (5 mmol L^−1^ ammonium acetate in water) and solvent B (acetonitrile). A gradient elution program was applied as follows: 95% A and 5% B at the initial condition; a linear gradient to 5% A and 95% B over 10 min; maintenance at 5% A and 95% B for 2 min; reversion to 95% A and 5% B within 0.1 min; and re‐equilibration for 2.9 min. The UPLC effluent was introduced into the LC‐MS/MS system for analysis. Data were acquired in data‐dependent acquisition (DDA) mode and processed using MS‐DIAL software. The ion source parameters were set as follows: sheath gas flow rate, 320 Arb; auxiliary gas flow rate, 40 Arb; source temperature, 350°C; and ion spray voltage floating (ISVF), 3400 V in positive ion mode and −3200 V in negative ion mode. TOF MS data were acquired over an m/z range of 85–1250. LC‐MS/MS analysis was performed by Biomisp (Wuhan, China).

### 5.9 Animal Experiment

Male C57BL/6J mice (6‐8 weeks old, 18–22 g) were purchased from Jiangsu Huachuang Xinnuo Pharmaceutical Technology Co., Ltd. Animals were housed under specific pathogen‐free (SPF) conditions and provided with unrestricted access to food and water. For establishment of the subcutaneous GC model, 1×10^6 YTN5 cells suspended in 200 µL PBS were injected subcutaneously into the left flank of each mouse. When tumor reached an average volume of approximately 50 mm^3^, mice were randomly assigned to four groups: control (Ctrl, *n* = 5, PBS), juice (*n* = 5, 10 mg kg^−1^), sup (*n* = 5, 10 mg kg^−1^), and PGNs (*n* = 5, 10 mg kg^−1^). Treatments were administered by intratumoral injection every other day for a total of seven doses. Tumor volume was calculated using the formula: *V* = 1/2×(longest diameter×shortest diameter^2). At the end of treatment, mice were euthanized, and tumor were excised and weighed. Major organs and tumor tissues were also collected for further analysis.

A subcutaneous flank tumor model was established as described above. Tumor‐bearing mice were randomly assigned to five groups (*n* = 5 per group): Ctrl (PBS), PGNs (10 mg kg^−1^), PGNs+ ferrostatin‐1 (Fer‐1, HY‐100579, MCE) (PGNs: 10 mg kg^−1^; Fer‐1: 3 mg kg^−1^), Gamma‐linolenic acid ( GLA, HY‐N7140, 10 mg kg^−1^), and GLA+Fer‐1 (GLA: 10 mg kg^−1^; Fer‐1: 3 mg kg^−1^). PGNs were administered via intratumoral injection every other day, Fer‐1 was delivered by daily intraperitoneal injection, and GLA was administered by daily oral gavage, for a total of seven doses. At the end of the treatment period, mice were euthanized, and tumors were excised and weighed. Major organs and tumor tissues were also harvested for subsequent analyses.

The subcutaneous flank tumor model was established as described above. Mice were randomly divided into five groups (n = 5 per group): Ctrl (PBS), PGNs‐L (5 mg kg^−1^), PGNs‐H (10 mg kg^−1^), anti‐PD‐1 (S0B0101, STARTER, 200 µg per mouse), and PGNs‐H plus anti‐PD‐1. PGNs were administered by intratumoral injection every other day for a total of seven doses. Anti‐PD‐1 antibody (200 µg per mouse) was administered by intraperitoneal injection every four days. At the end of the treatment period, mice were euthanized, and tumors were excised and weighed. All animal experiments were approved by the Institutional Review Board of Nanjing University of Chinese Medicine Affiliated Hospital (2024DW‐052‐02).

### 5.10 IHC Analysis

Tumor tissues from each group were collected, fixed, and embedded in paraffin, followed by sectioning for IHC analysis. Paraffin sections were deparaffinized, rehydrated, and subjected to antigen retrieval according to standard protocols. The sections were then incubated with primary antibodies against GPX4 (T56959, Abmart; 1:200) and Granzyme B (13588‐1‐AP, Proteintech, 1:500) at 4°C overnight. After washing, HRP‐conjugated secondary antibodies were applied and incubated at room temperature. Signal detection was performed using 3,3′‐diaminobenzidine, followed by counterstaining with Mayer's hematoxylin.

### 5.11 H&E Staining

Tissues from the liver, lung, heart, kidney, and spleen were harvested following euthanasia and immediately fixed in 10% neutral buffered formalin. After standard dehydration and paraffin embedding, serial sections (4 µm thickness) were prepared. Sections were subsequently deparaffinized, rehydrated through graded alcohols, and subjected to H&E staining according to established protocols. Following dehydration and clearing, slides were mounted with a neutral resin medium. Histopathological alterations were evaluated under a light microscope, and representative images were acquired for analysis.

### 5.12 In Vivo Fluorescence Imaging and Immunofluorescence Staining

Before administration, PGNs were labeled with the near‐infrared lipophilic carbocyanine dye DiR. Tumor‐bearing mice were intratumorally injected with DiR‐labeled PGNs, and whole‐body fluorescence images were acquired using an in vivo imaging system at 24 and 48 h after injection. For histological validation, tumors were harvested at the endpoint, embedded in OCT compound, and snap‐frozen. Frozen tumor sections were prepared, fixed, blocked, and counterstained with DAPI to visualize nuclei. Fluorescence images were then acquired using a confocal laser scanning microscope.

### 5.13 Single‐Cell RNA Sequencing of Tumor Tissue

For single‐cell RNA sequencing, tumor tissues were collected from Ctrl and PGN‐treated mice, with three independent biological samples per group. Fresh tumor tissues were minced, enzymatically dissociated with collagenase and DNase I, filtered through a 40 µm cell strainer, and resuspended in PBS containing 0.04% bovine serum albumin (BSA). Cell viability was assessed before library preparation.

Single‐cell suspensions were processed using the sCelluFlu‐A platform (CapitalBio Technology, Beijing, China). Approximately 10 000 viable cells from each sample were loaded for single‐cell encapsulation using the sCelluFlu‐A Single Cell Transcriptome Library Kit V1.2. Libraries were sequenced on the MGI DNBSEQ‐T7 platform using a paired‐end 150‐bp strategy.

Raw sequencing reads were processed using sCelluFluMatrix (v2.0.0) and aligned to the GRCm39/mm39 reference genome to generate unique molecular identifier (UMI) count matrices. Data analysis was performed using Seurat (v4.2.0). Each sample was initially subjected to quality control. Doublets were identified and removed using DoubletFinder (v2.0.3). Cells with fewer than 200 detected genes or mitochondrial transcript content >25% and genes detected in fewer than three cells were removed. The filtered datasets from all six samples were subsequently integrated, batch effects were corrected using Harmony, and downstream analyses were performed on the integrated dataset. After normalization and scaling, the top 2000 highly variable genes were used for principal component analysis (PCA). Cell clustering was performed using the first 30 principal components at a resolution of 0.6, and cell populations were visualized using t‐distributed stochastic neighbor embedding (t‐SNE).

T cells and epithelial cells were extracted from the integrated dataset and re‐clustered separately at a resolution of 0.1. Cell identities were assigned according to canonical lineage markers together with published single‐cell reference datasets. Differential gene expression analysis was performed using the Wilcoxon rank‐sum test, with significant genes defined by log_2_ fold change >0.25, *P* <0.01, and expression in >10% of cells in at least one comparison group. GO and KEGG enrichment analyses were performed using clusterProfiler (v4.4.4), and Circos plots were generated using Sangerbox. Detailed sequencing quality metrics are summarized in Supplementary (Table S1).

### 5.14 Western Blot Analysis

AGS and MKN‐28 cells and tumor tissues were lysed in RIPA buffer (P0013B, Beyotime) supplemented with 1 mM PMSF (ST506, Beyotime) and protease inhibitor cocktail (P1045, Beyotime). Protein concentrations were determined using a BCA Protein Assay Kit (P0009, Beyotime). Equal amounts of protein (20 µg per lane) were separated by 12% SDS–PAGE and transferred onto PVDF membranes (Millipore, Billerica). After blocking with 5% BSA for 1 h at room temperature, membranes were incubated overnight at 4°C with primary antibodies against GPX4 (T56959, Abmart; 1:1000), SLC7A11 (26864‐1‐AP, Proteintech; 1:1000), and GAPDH (5174S, CST; 1:1000). After three washes with TBST, the membranes were incubated with HRP‐conjugated goat anti‐rabbit secondary antibodies (RGAR001, Proteintech, dilution 1:10000) for 1 h at room temperature. Protein bands were visualized using an enhanced chemiluminescence (ECL) kit (BL523A, Biosharp) and detected with a ChemiDoc MP imaging system (Tanon). Band intensities were quantified using ImageJ software, and target protein expression was normalized to GAPDH.

### 5.15 GC Organoids Culture

GC organoids were established from tumor tissues obtained from the gastric body of patients at Jiangsu Provincial Hospital of Chinese Medicine. The collection and use of human gastric tumor specimens were approved by the Ethics Committee of institution under approval number (2025NL‐119‐01). Written informed consent was obtained from all participants, and all procedures were conducted in accordance with the Declaration of Helsinki. Tumor specimens were collected during surgical resection and washed vigorously three times with sterile cold PBS in 15 mL conical tubes. The tissues were then incubated in organoid digestion solution at 37°C for 1 h. Digestion was terminated by the addition of complete organoid culture medium, followed by centrifugation at 300 × g for 5 min to collect the cell pellet. The pellet was resuspended in Matrigel and seeded into 24 well plates. Organoids were maintained in complete culture medium, which was refreshed every 3–4 days until passaging.

### 5.16 Measurement of ROS, MDA and GSH Levels and BODIPY 581/591 C11 Fluorescence

AGS and MKN‐28 cells were treated with low‐dose PGNs (PGNs‐L, 40 µg mL^−1^) or high‐dose PGNs (PGNs‐H, 80 µg mL^−1^). For ferroptosis inhibition experiments, cells were pretreated with Fer‐1 (1 µm) for 1 h, followed by treatment with PGNs‐H for 24 h. Intracellular ROS levels were assessed by fluorescence microscopy following staining with 10 µm DCFH‐DA (S0033S, Beyotime). Lipid peroxidation was assessed using a Lipid Peroxidation MDA Assay Kit (S0131, Beyotime) according to the manufacturer's instructions. Briefly, samples and standards were prepared as specified, and absorbance was measured at 532 nm. MDA levels were normalized to total protein content and expressed as µmol mg^−1^ protein. GSH levels were measured using a GSH Assay Kit (A006‐2‐1, Nanjing Jiancheng) according to the manufacturer's instructions. BODIPY 581/591 C11 staining was performed by incubating AGS and MKN‐28 cells, as well as GC organoids, with 2 µm BODIPY 581/591 C11 (S0043S, Beyotime) in serum‐free medium at 37°C for 30 min in the dark. Samples were then washed with PBS and imaged by confocal laser scanning microscopy (Zeiss AiryScan, Germany).

### 5.17 T‐Cell Killing Assays In Vitro

Human peripheral blood mononuclear cells (PBMCs) were isolated using a lymphocyte separation medium (Sigma, USA). Peripheral blood was mixed with the separation medium at a 1:1 ratio and centrifuged at 800×*g* for 20 min with slow acceleration and deceleration. The PBMC layer was collected, washed twice with PBS (300 × *g* for 10 min), and re‐suspended in complete medium. Cells were allowed to rest for 5 h prior to T‐cell activation with anti‐CD3 (S0B0009, Starter) and anti‐CD28 (S0B0010, Starter) coated beads. GC cells were treated with varying concentrations of PGNs and subsequently co‐cultured with activated T cells at a 1:3 ratio. After 12 h, non‐adherent T cells were removed by PBS washing, and the viability of the remaining tumor cells was assessed. Human PBMCs were isolated from peripheral blood samples obtained from healthy donors. The use of human samples was approved by the Ethics Committee of Nanjing University of Chinese Medicine Affiliated Hospital under approval number: 2025NL‐119‐01, and written informed consent was obtained from all donors prior to sample collection. All procedures involving human samples were performed in accordance with the Declaration of Helsinki.

### 5.18 Flow Cytometry

Human peripheral blood‐derived T cells were activated with anti‐CD3/CD28 antibodies and treated with PGNs at the indicated concentrations. Cell viability was evaluated using the MTT assay. For flow cytometric analysis, cells were stained with fluorochrome‐conjugated antibodies against human CD3 (300320, BioLegend), CD8 (564116, BD Biosciences), and CD25 (302629, BioLegend) for surface‐marker analysis. For intracellular Granzyme B staining, cells were fixed, permeabilized, and stained with an anti‐human Granzyme B antibody (560212, BD Biosciences). IFN‐γ and TNF‐α concentrations in culture supernatants were quantified using a BD Cytometric Bead Array (CBA) Human Inflammation Kit (560484, BD Biosciences) or ELISA Kit (KE00146, KE00154, Proteintech ) according to the manufacturer's instructions. Samples were acquired on a BD FACSCanto flow cytometer.

AGS and MKN‐28 cells were pretreated with Fer‐1 (1 µm) for 1 h where indicated, followed by treatment with PGNs‐L, PGNs‐H, or PGNs‐H plus Fer‐1 for 24 h. Intracellular ROS was detected using DCFH‐DA (10 µm; S0033S, Beyotime), whereas lipid peroxidation was assessed using BODIPY 581/591 C11 (2 µm; S0043S, Beyotime). Cells were incubated with the corresponding probes at 37°C for 30 min in the dark, washed twice with PBS, and immediately analyzed by flow cytometry.

Single‐cell suspensions were prepared from freshly isolated mouse tumors by enzymatic digestion with collagenase IV and DNase I, followed by filtration through a 70 µm cell strainer. Cells were stained with a viability dye and fluorochrome‐conjugated antibodies against mouse CD45 (103116, BioLegend), CD3 (100216, BioLegend), CD4 (757772, BD Biosciences), and CD8a (752633, BD Biosciences). For intracellular staining, cells were fixed, permeabilized, and stained with antibodies against IFN‐γ (E07379‐1633, eBioscience), Granzyme B (563389, BD Biosciences), and Perforin (12‐9392‐82, Thermo Fisher Scientific). Flow cytometric analysis was performed by sequential gating on live singlet CD45^+^ leukocytes followed by CD3^+^CD8^+^T cells, and intracellular IFN‐γ, Granzyme B, and Perforin expression was quantified within the CD8^+^ T‐cell population. Samples were acquired on a Cytek flow cytometer and analyzed using FlowJo software.

### 5.19 FLIM Imaging and Data Analysis

PGNs and Fer‐1 interventions were performed as described above. After treatment, AGS and MKN‐28 cells were incubated with Flipper‐TR (CY‐SC020, 1 µm) in basal culture medium for 15 min. Live‐cell FLIM imaging was performed using a Leica TCS SP8 X confocal microscope equipped with a standard FLIM module. Flipper‐TR was excited with a 488 nm pulsed laser, and photon emission was collected through a 600 nm band‐pass filter. Fluorescence lifetime images were acquired under identical settings for all groups and analyzed using the FLIM module. Regions of interest were selected along the plasma membrane, and the mean fluorescence lifetime was quantified for each cell.

### 5.20 AFM and Data Analysis

AGS and MKN‐28 cells were seeded in 35‐mm culture dishes at 5000 cells per dish. After attachment, cells were treated with PGNs alone or with PGNs plus 1 µm Fer‐1 for 12 h at 37°C. Cells were then washed three times with 1 mL PBS and maintained at 37°C in a JPK dish heater during AFM measurement. Cell surface Young's modulus was determined using a Bruker NanoWizard Sense+CellHesion Module AFM equipped with a 100‐µm piezo scanner. Topographic imaging was acquired in QI Advance mode, and mechanical mapping was performed in contact mode with PFQNM‐LC‐A‐CAL probes (spring constant, 0.1 N m^−^^1^). The setpoint force was 300 pN. Raw data were analyzed using JPKSPM Data Processing software.

AGS and MKN‐28 cells were seeded into 35‐mm culture dishes at 5,000 cells per dish, subjected to the PGNs treatments for 24 h, and then cocultured with T cells for a further 12 h. Cells were washed three times with PBS, fixed in 4% paraformaldehyde for 10 min at room temperature, rinsed three times each with PBS and ultrapure water, and air‐dried at room temperature. Cell surface morphology was imaged using a Bruker NanoWizard Sense+CellHesion Module AFM equipped with a 100‐µm piezo scanner. Topographic imaging was acquired in QI Advance mode, and mechanical mapping was performed in contact mode with PFQNM‐LC‐A‐CAL cantilevers (spring constant, 0.1 N m^−^^1^). The setpoint force was 300 pN. Raw images were analyzed using JPKSPM Data Processing software.

### 5.21 Statistical Analysis

Statistical analyses were performed using GraphPad Prism 10.0. Comparisons between two groups were conducted using a two‐tailed unpaired Student's t‐test or the Mann‐Whitney U test. Multiple‐group comparisons were performed using one‐way analysis of variance (ANOVA) with Tukey's post hoc test. For experiments involving two independent variables, two‐way ANOVA was used. Paired data were analyzed using a paired two‐tailed Student's t‐test. A *p* value < 0.05 was considered statistically significant. Significance is denoted as *p* < 0.05 (* or #), *p* < 0.01 (** or ##); n.s. indicates no significant difference.

## Author Contributions

**Jian Wu**: conceptualization, methodology, and visualization. **Ruijuan Zhang** and **Yixin Duan**: investigation, data curation, and writing. **Shuqi Huo**, **Xiang Zhang**, and **Yibin Xing**: formal analysis and data interpretation. **Lingjing Ma**, **Tiran Jiang**, and **Xingyu Chen**: formal analysis and investigation. **Qingmin Sun**: funding acquisition, project administration, resources, and supervision. All authors reviewed and approved the final manuscript.

## Ethics Statement

All animal experiments were conducted in accordance with the ARRIVE guidelines and were approved by the Animal Ethics Committee of Nanjing University of Chinese Medicine Affiliated Hospital (2024DW‐052‐02).

## Conflicts of Interest

The authors declare no conflicts of interest.

## Supporting information




**Supporting File**: advs77900‐sup‐0001‐SuppMat.docx.

## Data Availability

All data supporting the principal findings of this study are included in the article. The raw data are available from the corresponding author upon reasonable request.
