# Supplementary material for: Platycodon grandiflorus–Derived Nanovesicles Sensitize Gastric Cancer to T Cell Killing via Ferroptosis‐Driven Membrane Mechanical Remodeling
Source: Adv Sci (Weinh). 2026 Sep 27:e77900. Online ahead of print. doi: 10.1002/advs.77900 (PMC13616398; doi:10.1002/advs.77900)
Supplement: Supplementary file 1 [file ADVS-9999-e77900-s001.docx]

**Supplementary material**

**Figure S1.** (A) Agarose gel electrophoresis showing nucleic acids extracted from PGNs. (B) SDS–PAGE profile of proteins extracted from PGNs. (C) Protein concentration of isolated PGNs (protein concentration, mg mL^-1^). (D) The purity of PGNs ( particles mg protein^-1^). (E) Particle size distribution and concentration (F) of PGNs determined by NTA after storing PGNs at varies condition. (G) Zeta potential of PGNs determined by NTA after storing PGNs at varies condition. (H) SDS–PAGE profile of proteins extracted from PGNs after storing PGNs at varies condition. (I) Representative results of EdU assays after treatment with juice, sup and PGNs for 24 h in MKN-28 and AGS cells. Scale bar = 100 μm. (J) Representative images of migration assay showing AGS and MKN-28 cells following treatment with juice, sup, and PGNs. Scale bar=100 µm. (K) Analyses of EdU assays results. (L) Quantification of cell migration. Data were presented as mean ± SD. Statistical significance was determined using one-way ANOVA followed by Tukey’s post hoc test. **P* < 0.05; ***P* < 0.01; ns, not significant.

**Figure S2.** (A, B) Representative total ion chromatograms of Juice samples acquired in the positive and negative-ion modes. (C, D) Representative total ion chromatograms of sup samples acquired in the positive and negative-ion modes. (E, F) Representative total ion chromatograms of PGNs samples acquired in the positive and negative-ion modes. (G, H) Volcano plots showing differential metabolites between PGNs and Juice or sup samples.

**Figure S3.** (A) Fluorescence microscopy images showing ROS levels in GC cells treated with PGNs-L and PGNs-H. Scale bar = 20 μm. (B) Quantitative analysis of ROS fluorescence intensity in AGS and MKN-28 cells. (C) Flow cytometric analysis of ROS fluorescence intensity in AGS and MKN-28 cells. (D) Quantification of the mean fluorescence intensity (MFI) of ROS in AGS and MKN-28 cells. (E) MDA levels in cells treated with PGNs-L and PGNs-H. (F) Intracellular GSH levels in cells treated with PGNs-L and PGNs-H. (G) Fluorescence staining of the lipid peroxidation sensor BODIPY 581/591 C11 in GC cells treated with PGNs-L and PGNs-H. Scale bar = 20µm. (H) Quantitative analysis of lipid peroxidation in AGS and MKN-28 cells. (I) Representative flow cytometric analysis of lipid peroxidation in AGS and MKN-28 cells. (J) Lipid peroxidation was quantified as the MFI of oxidized C11-BODIPY detected in the FITC channel and normalized to the control group. (K, L) Western blot analysis of the proteins SLC7A11 and GPX4 in cells treated with PGNs-L and PGNs-H. Data were presented as mean ± SD. Statistical significance was determined using one-way ANOVA followed by Tukey’s post hoc test. **P* < 0.05; ***P* < 0.01; ns, not significant.

**Figure S4.** (A) Quantitative analysis of ROS fluorescence intensity in AGS and MKN-28 cells treated with PGNs alone or PGNs combined with Fer-1. (B) Flow cytometric analysis of ROS fluorescence intensity in AGS and MKN-28 cells treated with PGNs alone or PGNs combined with Fer-1. (C) Quantification of the MFI of ROS in AGS and MKN-28 cells. (D) Quantitative analysis of lipid peroxidation in GC organoids treated with PGNs in the presence or absence of Fer-1. (E) Quantitative analysis of lipid peroxidation in AGS and MKN-28 cells treated with PGNs alone or PGNs combined with Fer-1. (F) Representative flow cytometric analysis of lipid peroxidation in AGS and MKN-28 cells treated with PGNs alone or PGNs combined with Fer-1. (G) Lipid peroxidation was quantified as the MFI of oxidized C11-BODIPY detected in the FITC channel and normalized to the control group. Data were presented as mean ± SD. Statistical significance was determined using one-way ANOVA followed by Tukey’s post hoc test. **P* < 0.05; ***P* < 0.01; ns, not significant.

**Figure S5.** (A) Dot plot showing the expression of representative marker genes for each cell type. (B) Using the T cells, B cells, NK cells as the reference cell group ( upper heat map ). And the Epithelial cells ( lower heat map ) as the observation cell group for CNV analysis and the results of inferCNV. The horizontal axis represents the 20 axes arranged in order, and the different colors on the vertical axis correspond to different cell subgroups. (C) t-SNE plot of the classification of malignant and non-malignant cells. (D) t-SNE plot of malignancy levels in epithelial cell subgroups. (E) Violin plot showing the expression of four malignant cell representative genes with differential expression between malignant and non-malignant cells. (F) Violin plot showing CNV scores of malignant cells after PGNs treatment. (G) GO enrichment analysis of molecular function for down-regulated genes in malignant cells after PGNs treatment. Data were presented as mean ± SD. Statistical significance was determined using an unpaired two-tailed Student’s t-test. **P* < 0.05, ***P* < 0.01; ns, not significant.

**Figure S6.** (A) Dot plot showing the expression of representative marker genes for T cell type. (B) Heatmap showing scaled expression of dynamic genes along the pseudotime. Rows of the heatmap represent genes that show dynamic changes along the pseudotime, and these genes were clustered into six groups according to their expression pattern along the pseudotime. (C) The color gradient from dark to light indicates the progression of pseudotime. Pseudo-time trajectory analysis of T cells based on groups. (D) Flow cytometric quantification of granzyme B level in T cells co-culture with GC cells. (E) Quantitative analysis of granzyme B. (F) T cells were co-cultured with GC cells pretreated with increasing concentrations of PGNs, and GC cell viability was assessed by MTT assay. (G) MTT analysis of GC cell viability following treatment with increasing concentrations of PGNs and co-culture with T cells, with or without Fer-1. Data were presented as mean ± SD. Statistical significance was assessed using one‑way ANOVA with Tukey’s post hoc test for single‑factor experiments, and two‑way ANOVA with Sidak’s multiple‑comparisons test for comparisons between different groups at each concentration level. The interaction between concentration and group was evaluated within the two‑way ANOVA model. *, #*P* < 0.05; **, ##*P* < 0.01; ns, not significant. * indicates between-group comparisons at the same concentration, whereas # indicates within-group comparisons across different concentrations.

**Figure S7.** (A) The t-SNE projection of Tc cells subclusters in Ctrl and PGNs group. (B) The proportion of Tc cell type in Ctrl groups and PGNs groups. (C) Dot plot showing the expression of representative marker genes for Tc cell type. (D) Violin plots showing the expression of representative differentially expressed Tc-cell genes between the Ctrl and PGNs groups. (E) MTT analysis of T cell viability following treatment with increasing concentrations of PGNs. (F) Representative flow cytometry plots showing the proportion of CD8⁺CD3⁺ T cells after treatment with Ctrl, PGNs-L, or PGNs-H. (G) Quantification of CD8⁺CD3⁺ T cells in the indicated groups. (H) Representative flow cytometry plots showing CD25 expression in CD3⁺ T cells and CD8⁺/CD8⁻ T-cell subsets after PGNs treatment. (I) Quantification of CD25⁺CD3⁺ T cells. Quantification of CD25⁺CD8⁻ T cells (J) and CD25⁺CD8⁺ T cells (K) in the indicated groups. ELISA-based quantification of TNF-α (L) and IFN-γ (M) levels in the culture supernatants after treatment with Ctrl, PGNs-L, or PGNs-H. Data were presented as mean ± SD; Statistical significance was determined using an unpaired two-tailed Student’s t-test for two-group comparisons, and one‑way ANOVA with Tukey’s post hoc test for multiple-group comparisons. **P* < 0.05, ***P* < 0.01; not significant.

**Figure S8**. (A) Body weight changes of tumor-bearing C57BL/6J mice during treatment with Ctrl, PGNs-L, PGNs-H, anti-PD-1, or PGNs-H combined with anti-PD-1. (B) MTT analysis of YTN5 cell viability following treatment with increasing concentrations of PGNs. (C) Lymphocytes were first gated based on FSC-A and SSC-A, followed by singlet selection using FSC-A and FSC-H. Dead cells were excluded using a viability dye, and live CD45⁺ immune cells were subsequently gated. T cells were identified as CD45⁺CD3⁺ cells, and CD4⁺ and CD8⁺ T-cell subsets were further defined within the CD3⁺ T-cell population. For analysis of cytotoxic effector function, CD8⁺ T cells were gated and the frequencies of Granzyme B⁺CD8⁺ T cells, IFN-γ⁺CD8⁺ T cells, and Perforin⁺CD8⁺ T cells were quantified. Data were presented as mean ± SD. Statistical significance was determined using one-way ANOVA followed by Tukey’s post hoc test. **P* < 0.05, ***P* < 0.01; not significant.

**Supplementary Table 1. Quality-control metrics for individual samples used in single-cell RNA sequencing**

| Sample | Group | Cells after QC | Median genes | Median UMIs | Median mitochondrial ratio | Median hemoglobin ratio | Median ribosomal ratio |
| --- | --- | --- | --- | --- | --- | --- | --- |
| Ctrl-1 | Ctrl | 10022 | 766 | 1574.5 | 8.75% | 0.00% | 22.21% |
| Ctrl-2 | Ctrl | 5001 | 1027 | 2219 | 7.25% | 0.00% | 19.53% |
| Ctrl-3 | Ctrl | 9360 | 813 | 1614.5 | 6.17% | 0.00% | 18.95% |
| PGN-1 | PGN | 10284 | 809 | 1607 | 5.47% | 0.00% | 19.62% |
| PGN-2 | PGN | 9300 | 817 | 1734 | 7.79% | 0.00% | 22.23% |
| PGN-3 | PGN | 9997 | 1091 | 2210 | 2.70% | 0.00% | 15.71% |

**Note:** Tumor tissues from three independent biological samples per group were subjected to single-cell RNA sequencing. The values shown represent quality-control metrics for each individual sample after preprocessing. Cells with fewer than 200 detected genes or mitochondrial transcript content >25% were excluded. Genes detected in fewer than three cells and doublets identified using DoubletFinder (v2.0.3) were also removed prior to downstream analyses. Batch effects among the six samples were corrected using the Harmony algorithm before data integration, clustering, and differential expression analyses. All six samples met the predefined quality-control criteria for downstream analyses. A total of 53,964 high-quality cells were retained for downstream analyses.
